# Advances in CircRNAs in the Past Decade: Review of CircRNAs Biogenesis, Regulatory Mechanisms, and Functions in Plants

**DOI:** 10.3390/genes15070958

**Published:** 2024-07-21

**Authors:** Dongqin Zhang, Yue Ma, Misbah Naz, Nazeer Ahmed, Libo Zhang, Jing-Jiang Zhou, Ding Yang, Zhuo Chen

**Affiliations:** 1 Key Laboratory of Green Pesticide and Agricultural Bioengineering, Ministry of Education, Guizhou University, Guiyang 550025, China; zhang15186832671@163.com (D.Z.); misbahnaz.ray@yahoo.com (M.N.); drnazeerento@gmail.com (N.A.); lbzhang@gzu.edu.cn (L.Z.); xlh22@cam.ac.uk (J.-J.Z.); dyangcau@126.com (D.Y.); 2 College of Agriculture, Guizhou University, Guiyang 550025, China; yma3@gzu.edu.cn; 3 Medical Research Council Mitochondrial Biology Unit, University of Cambridge, Cambridge CB2 0XY, UK

**Keywords:** circRNAs, biogenesis, regulatory mechanisms, growth and development, stress response

## Abstract

Circular RNA (circRNA) is a type of non-coding RNA with multiple biological functions. Whole circRNA genomes in plants have been identified, and circRNAs have been demonstrated to be widely present and highly expressed in various plant tissues and organs. CircRNAs are highly stable and conserved in plants, and exhibit tissue specificity and developmental stage specificity. CircRNAs often interact with other biomolecules, such as miRNAs and proteins, thereby regulating gene expression, interfering with gene function, and affecting plant growth and development or response to environmental stress. CircRNAs are less studied in plants than in animals, and their regulatory mechanisms of biogenesis and molecular functions are not fully understood. A variety of circRNAs in plants are involved in regulating growth and development and responding to environmental stress. This review focuses on the biogenesis and regulatory mechanisms of circRNAs, as well as their biological functions during growth, development, and stress responses in plants, including a discussion of plant circRNA research prospects. Understanding the generation and regulatory mechanisms of circRNAs is a challenging but important topic in the field of circRNAs in plants, as it can provide insights into plant life activities and their response mechanisms to biotic or abiotic stresses as well as new strategies for plant molecular breeding and pest control.

## 1. Introduction

Circular RNA (circRNA) is a distinct type of endogenous non-coding RNA that differs from normal linear RNA. CircRNAs are frequently generated through covalent linkage of the 5′ end of an upstream exon to its 3′ end, or to the 3′ end of a downstream exon [1,2,3]. With the development of high-throughput sequencing technology and bioinformatics methods, circRNAs were first discovered in *Arabidopsis thaliana* in 2014 [4]. CircRNAs have been found in plants such as rice (*Oryza sativa* L.), wheat (*Triticum aestivum* L.), barley (*Hordeum vulgare* L.), maize (*Zea mays* L.), soybean (*Glycine max*), potato (*Solanum tuberosum* L.), tomato (*Solanum lycopersicum*), bell pepper (*Capsicum annuum* L.), watermelon (*Citrullus lanatus* Thunb.), cucumber (*Cucumis sativus* L.), cotton (*Gossypium hirsutum* L.), kiwifruit (*Actinidia chinensis* Planchon), tea (*Camellia sinensis*), sea buckthorn (*Hippophae rhamnoides* L.), apple (*Malus baccata* (L.) Borkh.), and pear (*Pyrus betulifolia* Bunge) [5,6,7,8,9,10,11,12,13,14,15,16,17,18,19,20]. The reported quantities of circRNAs substantially vary across species and tissues because of differences in the tissues, sequencing depth, and (or) bioinformatics methods applied. For example, 12,037 and 6012 circRNAs have been identified from the root of rice and the leaf of *A. thaliana*, respectively [21]. Moreover, 1256, 1064, 912, 904, and 1080 circRNAs have been, respectively, identified from the roots, phloem, leaves, flowers, and fruits of apple [19]. With the development of technology, studies are increasingly indicating that the expression of circRNAs exhibits cell, tissue, and developmental stage specificity [22].

According to previous studies, circRNAs originate from exons, introns, or intergenic regions of the genome [23]. However, most circRNAs are produced from one or more exons of protein-coding genes, which are called parental genes or host genes of circRNAs [24]. CircRNAs are usually produced through splicing mechanisms and are formed as closed-loop structures comprising one or more exons [24]. The splicing mechanism of circRNAs is a characteristic formation mechanism that differs from the splicing method of linear RNAs [1]. Studies increasingly support two main models of the origin and biogenesis of circRNAs: the direct backsplicing model and exon jumping model (also known as the lariat precursor model) [1,3,25,26]. Moreover, circRNA biogenesis is regulated by specific cis-regulatory elements and trans-acting factors [27]. CircRNAs have been suggested to exert their biological functions primarily by acting as miRNA sponges, regulating the translation of target genes of miRNAs, influencing the transcription of their parental genes, or affecting polypeptide translation by interacting with proteins [28,29]. Databases and tools for analyzing plant circRNAs remain under development and continue to improve. Many circRNAs have been identified and found to exhibit tissue-specific expression [25]. These circRNAs affect plant growth and development, and play important regulatory roles in responses to biotic and abiotic stresses [28]. 

In this review, we describe circRNAs’ detailed biogenesis mechanisms, types, and biological functions in plants. We further discuss the importance of circRNAs in plant growth, development, fruit formation, aging, and responses to biotic and abiotic stresses. Regarding the applications of circRNAs in botany, we discuss the mechanisms of circRNAs with reference to recent advances in the fields of medicine and zoology. With the continuing progress in technology and in-depth research, we predict that research on circRNAs in the fields of gene regulation, molecular breeding, and pest control will attract broader attention (Table 1). Research on circRNAs is likely to provide new perspectives that can reveal the complexity of plant life activities and the mechanisms underlying pest outbreaks, and provide useful information for developing new strategies for pest control.

## 2. Classification of circRNAs

On the basis of previous studies, circRNAs can be classified according to their sequence sources and biogenesis mechanisms. Types include exonic circRNAs (EcircRNAs), intronic circRNAs (ciRNAs), exon-intron circRNAs (EIciRNAs), and intergenic circRNAs [30,31]. The different types of circRNAs exhibit differing expression patterns and biological functions. The discovery and functional understanding of circRNAs remain nascent [26]. Therefore, further study is needed on classifying circRNAs and their functions and on improving the classification system for circRNAs.

### 2.1. Exonic circRNAs

EcircRNAs are circular RNAs formed from one or more exonic sequences [32]. This type of circRNA is formed through covalent linkage of its 5′ and 3′ ends through “head-to-tail” splicing [22]. EcircRNAs were initially considered a byproduct of RNA transcription and splicing [33]. With the widespread application of RNA-seq technology and the rapid development of molecular biology technology, EcircRNAs are increasingly being identified. EcircRNAs are predominantly cytoplasmic and regulate gene transcription and expression by influencing translation, RNA interference, or sequestration of RNA-binding proteins (RBPs) [34].

### 2.2. Intronic circRNAs

CiRNAs are intronic sequences that form RNA loops [35]. CiRNAs are generated through release of the exon at the 5′ end and attack of the 3′ splice site by the terminal 3′-OH. Simultaneously, exons at the 3′ end are released [22]. Unlike EcircRNAs, ciRNAs are found primarily in the nucleus [36]. Some abundantly expressed ciRNAs have been found to act as “molecular sponges” for the TAR DNA-binding protein 43 (TDP-43) and other RBPs in the nucleus [36]. In some cases, ciRNAs regulate gene expression. The knockdown of ciRNAs decreases the expression of their parental genes, particularly at medical region [30]. For example, the knockdown of circRNA-EIF3J in human cervical carcinoma HeLa cells significantly decreases the expression of *Eukaryotic translation initiation factor 3J* (*EIF3J*) mRNA [37]. Ci-ankrd52 is an abundant ciRNA whose knockdown significantly decreases the expression of *Ankyrin repeat domain 52* (*ANKRD52*) mRNA. Meanwhile, ci-ankrd52 accumulates primarily in the nucleus, thus promoting the transcription of *ANKRD52* through the cis-regulatory action of RNA polymerase II (Pol II) [36].

### 2.3. Exon-Intron circRNAs

EIciRNAs are circRNAs containing both exons and introns [22]. EIciRNAs form primarily by retaining introns when the upstream and downstream of exons are backspliced [24]. EIciRNAs are present primarily in the nucleus [36]. The nuclear localization of EIciRNAs and their association with Pol II suggest their potential involvement in transcriptional regulation [37]. The retained intron 5′ of EIciRNAs contains a key site for binding U1 small nuclear RNA (U1 snRNA). Through this binding site, EIciRNAs bind U1 snRNA and form a complex that enhances the transcription of EIciRNA parent genes [37]. EIciRNAs may have other regulatory roles and mechanisms, and further in-depth studies are needed to reveal the specific roles of EIciRNAs in gene regulation and cellular processes.

### 2.4. Intergenic circRNAs

Intergenic circRNAs are generated from intergenic regions of the genome, i.e., regions between two genes [38]. Intergenic circRNAs can be found in both the nucleus and the cytoplasm, and may be involved in gene regulation and expression [39]. Genome structure analyses have indicated that the distribution of intergenic circRNAs among genes exists. One study identified circ7379, an intergenic circRNA derived from in chr14:35020919-35024118 with a splicing length of 3199 nucleotides (nt) [39]. Another study found that intergenic circRNAs account for 24.4% of the total circRNAs in tomato fruits with or without chilling treatment [11]. However, the specific functions and regulatory mechanisms of intergenic circRNAs require further research to clarify the network architecture and mechanisms of gene regulation.

## 3. Biogenesis of circRNAs

**Figure 1:**
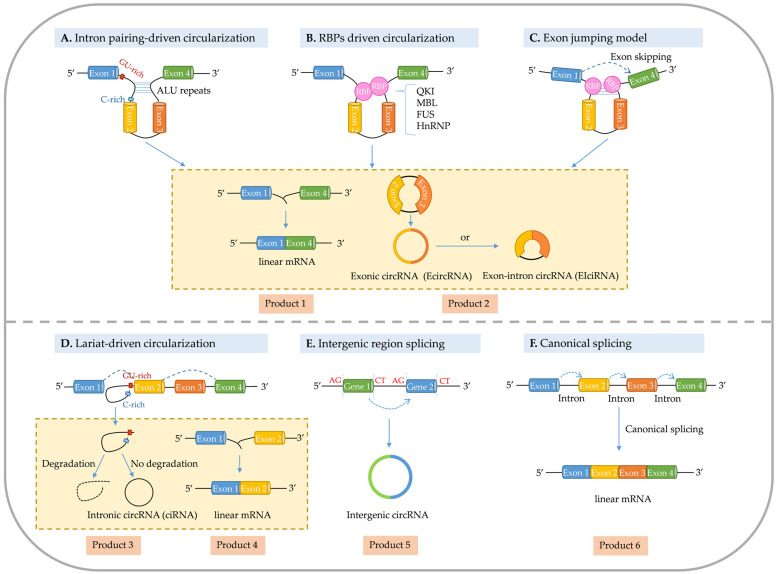
The biogenesis mechanism of circRNAs. ALU repeats, arthrobacter luteus repeats; HnRNP, heterogeneous ribonucleoprotein; FUS, fused in sarcoma; MBL, muscleblind; RBP, RNA-binding protein; QKI, quaking.

Precursor messenger RNAs (pre-mRNAs) produce mRNA and lariat introns through canonical splicing [40]. However, circRNAs are circular single-stranded RNAs formed by alternative splicing (AS) of mRNA [26]. The origins of circRNAs differ. Two main routes have been reported to drive circRNA cyclization: the direct backsplicing model and the exon jumping model (Figure 1) [41].

### 3.1. Direct Backsplicing Model

The direct backsplicing model is also known as the mechanism of intron pairing-driven cyclization [42]. Although backsplicing is considered a form of AS, its molecular mechanism differs from that of linear AS [24]. Direct backsplicing is completed through complementary base pairing of introns flanking exons; the exon 5′-splice donor site is combined with the upstream 3′-splice acceptor site, and finally the exons are cyclized to form circRNAs [43]. Many studies have shown that direct backsplicing is regulated by cis-regulatory elements and trans-acting factors [27]. Thus, the direct backsplicing model can be subdivided into intron-pairing driven cyclization and trans-acting factor driven cyclization [27,44]. Cyclization induced by intron pairing is completed through direct insertion of reverse complementary sequences (such as the Alu sequence) and by removal or retention of introns, thus ultimately yielding EIciRNAs or EcircRNAs [45]. RBPs bind specific motifs of flanking introns and subsequently initiate cyclization. Splicing factors containing quaking (QKI) and muscleblind (MBL), as well as proteins such as heterogeneous ribonucleoprotein (HnRNP) and serine/arginine-rich (SR) proteins were found to be involved in circRNA biogenesis [46]. The detailed mechanisms of circRNA generation are described below.

#### 3.1.1. Intron Pairing Drives Cyclization

The cyclization pattern driven by intron pairing is caused primarily by two flanking intron sequences of cyclized exons and is completed through the RNA secondary structure or the restriction endonuclease recognition sequence in the intron (Alu sequence) connecting the 5′-splicing donor site of the precursor mRNA to the 3′ splicing acceptor site via reverse complementary pairing, thus ultimately forming EcircRNAs or EIciRNAs [47]. The miniature introns and short reverse repeats of ~30–40 nucleotides in length, such as Alu elements, are sufficient to cyclize regulated exons. This process requires of intron repeats, thus bringing splicing sites into proximity and promoting base pairing cyclization. The intron repeat sequence and exon sequence must cooperate to achieve cyclization of RNA in coordination with a functional 3′ end processing signal. However, not all repeat sequences support cyclization. Hairpin structures between repeats may increase the stability of the sequence and inhibit RNA cyclization [47]. For example, in transient over-expression of circRNA in grapes (*Vitis vinifera* L.), the length of the flanking intron affects the efficiency of cyclization of the RNA: longer flanking intron lengths are associated with higher cyclization formation efficiency. A flanking intron repeat sequence of at least 40 nt length was found to be sufficient for exon cyclization [48].

Compared with animal circRNAs, most plant circRNAs have fewer repeat sequences and reverse complementary sequences in the intron sequences flanking EcircRNAs. For example, 6074 and 5152 EcircRNAs were detected in rice and *Arabidopsis*, respectively. Reverse complementary sequences with lengths > 15 nt between flanking introns were found in only forty-six and one EcircRNAs, respectively [21]. Similarly, only 20 of 2354 circRNAs were found to contain complementary sequences greater than or equal to 18 nt in their flanking intron sequences in rice [5]. Of the 2494 circRNAs in soybean, only 2.7% of the sequences of the introns flanking EcircRNAs contain reverse complementary sequences [49]. In maize, 17.3% of flanking regions in circRNAs were found to contain sequences associated with interspersed nuclear element 1-like elements (LLEs) and their reverse complementary pairs. The sequences associated with LINE1-like elements (LLEs) and their reverse complementary pairs (LLERCPs) are significantly enriched in the regions flanking circRNAs. When the number of LLERCPs increases, the accumulation of circRNAs significantly varies, whereas the abundance of circRNAs of linear transcripts decreases. Furthermore, genes with LLERCP-mediated circRNAs are enriched in loci associated with phenotypic variation in maize. Thus, circRNAs are likely to be associated with the regulation of phenotypic variation caused by LLERCPs [8]. In moso bamboo (*Phyllostachys edulis*), only 10 inverse complementary sequences > 30 nt in length were detected from the flanking intron sequences of 720 EcircRNAs [50]. Only a few circRNAs were found to be regulated by flanking reverse complementary sequences.

#### 3.1.2. Trans-Acting Factors Drive Cyclization

Several studies have shown that Alu elements on complementary flanking sequences with introns have important roles in cyclization [34,45]. Although the Alu element at introns is a key regulator of circRNA biogenesis, recent studies have indicated that many trans-acting factors have positive or negative effects on circRNA biogenesis [34,51]. Another route of circRNA biogenesis is the cyclization mode driven by trans-acting factors, which bind the specific sequences of flanking introns through RBPs or several splicing factors, thus forming a bridge bringing splicing donor and splicing recipient sites into proximity sufficient for formation of circRNAs [51]. In addition, RBPs promote cyclization by stabilizing complementary sequences or inhibiting canonical splicing [52]. This cyclization pattern is similar to the pathway driven by intron pairing, except that the RBP-induced splicing sites are closer to each other than to the bases from complementary sequences [42].

Two tissue-specific splicing factors, MBL and QKI, induce the cyclization of the sequences that bind RBPs [44,51]. MBL was found to promote the biosynthesis of circMbl by binding flanking introns. Elevated MBL expression decreases normal splicing and promotes reverse splicing of linear mRNAs, thus inducing circMbl production [44]. The selective splicing factor QKI binds specific sequences on flanking introns, thereby bringing spliceosome sites on pre-mRNAs into proximity and leading to circRNA formation [51]. In *A. thaliana*, five proteins with high sequence homology to QKI have been found to be involved in pre-mRNAs processing and play important roles in regulating plant flowering and hormone signaling [53,54]. These five proteins may have similar functions to QKI, for example, in circRNA production [55]. Although proteins homologous to QKIs have been identified in *A. thaliana*, their potential functions in circRNA generation remain to be investigated.

Other trans-acting factors have been implicated in circRNA biogenesis, such as various SR proteins, HnRNP, and tissue-specific splicing proteins or factors (such as RNA-binding motif protein 20 (Rbm20); fused in sarcoma (FUS), which encodes an RNA-binding protein with diverse roles in transcriptional activation and RNA splicing; and DEAH-box helicase 9 (DHX9)). These proteins or factors may promote or inhibit circRNA formation [56]. HnRNP and SR proteins work together with the repeat sequences on intron in regulating the cyclization of the *laccase2* gene in *Drosophila melanogaster* [56]. FUS was found to bind the selective splice junction sites on flanking sequences from introns and to regulate circRNA synthesis [57]. Although most RBPs promote circRNA generation, some RBPs inhibit circRNA formation. For example, adenosine deaminase acting on RNA 1 (ADAR1) edits adenosine nucleosides to hypoxanthine nucleosides (adenosine-to-inosine (A-to-I) editing) by binding double-stranded RNA, whereas the Alu element is the main binding target site for ADAR, and A-to-I editing interferes with the stability of paired elements, such as Alu, thus inhibiting circRNA formation [58]. CircRNAs originating from a specific region within the *titin* gene are significantly inhibited by the knocking of *Rbm20* gene [59]. In addition, DHX9, with functional domains for RNA-binding and helicase activity, binds a reverse-complementary Alu element and subsequently unravels reverse-complementary Alu sequences, thereby inhibiting circRNA production [60].

### 3.2. Exon Jumping Model 

The exon jumping model is also known as the mechanism of lariat-driven cyclization [1]. This model focuses on the partial folding occurring during pre-mRNA transcription, during which exons appear to “jump” as the RNA folds, thus resulting in the formation of an intermediate lasso structure of exons and introns at the spanned site. Subsequently, in reverse splicing within the lasso structure, the introns may or may not be excised by the spliceosome, thus forming EcircRNAs or an EIcircRNAs, respectively [43]. RNA-seq and RT-qPCR experiments have indicated that exon skipping may induce circRNA formation, in agreement with prior research [61,62,63,64]. The strong coupling between exon jumping and circRNA generation was demonstrated by studying the *mitochondrial ribosomal protein S16* (*mrps16*) gene in *Schizosaccharomyces pombe* [65]. The mechanism was speculated to involve a splicing action for exons 1 and 3 of the *mrps16* gene, release of the intron lasso containing exon 2, and subsequent resplicing joining the origin and endpoint of exon 2, thus forming circRNAs subsequently. CircRNAs accumulate in the cell, and the double lasso is rapidly degraded [65].

In addition, the intron lasso structure formed during splicing is rapidly degraded under the action of debranching RNA lariats 1 [35,66]. However, some introns containing key nucleic acid sequences cannot be degraded by debranching enzymes after splicing, and the introns can form intron-derived circular RNAs (ciRNAs) [36]. The formation of ciRNAs depends on the 7 nt GU-rich element near the 5′ splice site and the 11 nt C-rich element near the branching site, which forms a lariat intron via transcription by Pol II, and is ultimately cyclized through covalent linkage to the 2′–5′ phosphodiester bond. Subsequently, the excess sequence is degraded from the 3′ end of the intron to the branching site [36]. Lariat structures widely exist in *Arabidopsis*, tomato, rice, and corn, and many circRNAs driven by lariat structures are being identified [67].

In summary, factors associated with circRNA formation include specific intron sequences, RBP binding sites, and exon jumping [68]. However, current understanding remains limited, and further studies are needed to explore how these components regulate circRNA biogenesis. Future studies may indicate the mechanisms of circRNA formation of more RBPs and spliceosomes. A deeper understanding of the expression and functional changes in these regulatory elements in different tissues and developmental stages is necessary. In-depth understanding of circRNA formation mechanisms would reveal the important functions and regulatory mechanisms of circRNAs in biological processes.

## 4. Interaction Mechanisms of circRNAs with Other Biological Macromolecules

**Figure 2:**
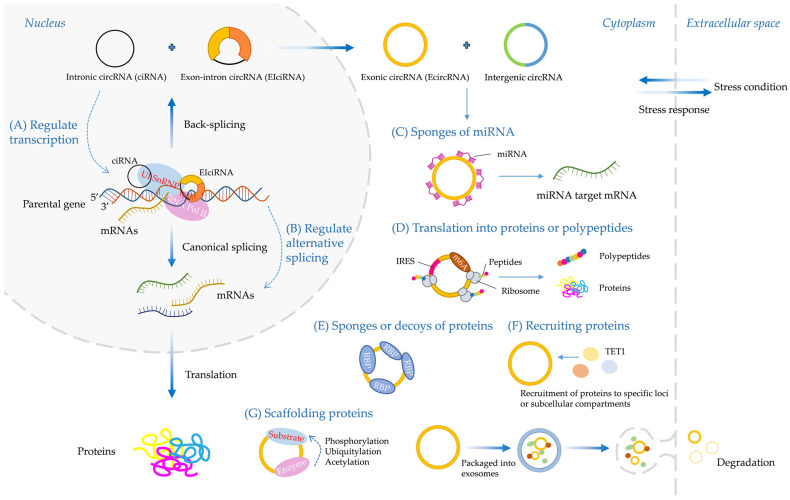
The biological function of circRNAs. IRES, internal ribosome entry site; m6A, N6-methyladenosine; RBP, RNA-binding protein; TET1, ten-eleven translocation.

CircRNA includes types such as ciRNA, EIciRNA, EcircRNA, and intergenic circRNAs. Research indicates that they possess various biological functions, such as regulating gene transcription, regulating alternative splicing of mRNAs, acting as miRNA sponges, influencing the translation of proteins or peptides, functioning as protein sponges or decoys, recruiting proteins, and serving as scaffolding protein, which were summarized below (Figure 2, Table 2).

### 4.1. CircRNAs and Their Parental Genes

During the splicing of precursor mRNAs, circRNAs produced by backsplicing competitively regulate the splicing of their linear transcripts, thereby affecting the expression of their parental genes [44]. CircRNAs regulate expression of their parental genes through various mechanisms, such as transcription and splicing regulation, miRNA sponges, mRNA traps, translational regulation, and post-translational modification [87]. Some studies have shown that cyclization of more exons during circRNA biogenesis results in fewer exons present in the processed mRNA [45,61]. The second exon of the *MBL* gene, which is known as the splicing factor, is cyclized, thus forming circMbl. The flanking intron of circMbl has a conserved *MBL* gene binding site, which binds the *MBL* gene. The regulation of *MBL* expression affects the generation of circMbl, and changes in circMbl expression competitively regulate classical splicing, thereby affecting the expression of *MBL* [44].

Although most circRNAs are located in the cytoplasm, ciRNAs are produced from processed intron lariats, and EIciRNAs with retained introns are produced from reverse splicing. Both circRNAs and EIciRNAs are present in the nucleus [36,37,41]. The R-loop—a three-stranded nucleic acid structure containing a DNA–RNA heterodimer and a single-stranded DNA molecule—affects DNA replication, repair, and transcription [88]. Ci-ankrd52 is produced from the second intron of the precursor mRNA of *ANKRD52* and is localized primarily in the nucleus [36]. Ci-ankrd52 has a unique secondary structure that allows it to form a more stable R-loop with its parental gene. Moreover, RNase H1 mediates the degradation of ci-ankrd52, which facilitates transcriptional elongation by Pol II [35]. Here, we take the pathogenic mechanism of human disease as an example. CircSMARCA5 binds its parental gene and forms an R-loop, thus leading to transcriptional pausing at exon 15 of *SMARCA5*. The expression of circSMARCA5 leads to downregulation of the *SMARCA5* gene. Overexpression of circSMARCA5 is sufficient to increase the sensitivity of breast cancer cells to the antitumor agents, cisplatin and bleomycin [70]. In addition, circEIF3J and circPAIP2 from EIciRNAs bind U1 snRNP and form a complex, thus decreasing transcript levels of *EIF3J* and *PAIP2* by knocking down circEIF3J and circPAIP2 [37]. CircPABPN1 segregates Human antigen R (HuR) protein, thereby acting as a decoy for HuR and impairing the translation of *Poly(A)-binding protein nuclear 1* (*PABPN1*) mRNA [89]. Together, these studies have suggested that some nuclear-localized circRNAs regulate gene expression at both the transcriptional and splicing levels.

CircRNAs also regulate the expression of host genes through epigenetic modifications [69,90]. N6-methyladenosine (m6A) is an important methylation modification of adenine [91]. Many m6A-modified circRNAs (m6A-circRNAs) have been found in human cells and determined to affect the stability of host genes [92]. FECR1 circRNA, derived from three exons of the *Friend leukemia virus integration 1* (*FLI1*) gene, specifically binds the *FLI1* promoter region and recruits demethylase ten-eleven translocation 1 (TET1), thereby inducing demethylation in the region and regulation of the expression of target genes [69]. In addition, the first m6A-binding protein *epithelial cell transforming sequence 2 oncogene* (*ECT2*) was identified in *A. thaliana*. *ECT2* regulates modification of the 3′ untranslated region in the nucleus and the stability of linear RNA in the cytoplasm [93].

### 4.2. CircRNAs and miRNAs

#### 4.2.1. CircRNAs Act as miRNA Sponges

Several studies have reported that circRNAs act as miRNA sponges regulating the expression of target genes by binding miRNAs [23,94]. A circRNA binds one or more miRNAs through the binding sites of multiple miRNAs in a circular sequence [24]. For example, cerebellar degeneration-related protein 1 transcript (CDR1as), also known as ciRS-7, originating from human and mouse brains, is highly expressed, and strongly represses miR-7 activity and increases the expression of miR-7 target mRNAs through 63 conserved miR-7 binding sites [23]. CircSRY interacts with miR-138 through its 16 miR-138 binding sites and therefore acts as a miR-138 sponge, thus regulating the expression of AGO2 proteins in HEK293 cells [94]. CircITCH binds miR-214 and miR-22-3p, and promotes the expression of *Itchy E3 Ubiquitin Protein Ligase* (*ITCH*) and *Casitas B lineage lymphoma* (*CBL*) genes, thereby regulating the WNT/β-catenin pathway [95,96]. CircITCH also promotes the expression of *Phosphatase and tensin homologue* (*PTEN*) and *RAS P21 Protein Activator 1* (*RASA1*), which participate in the PI3K/AKT and MAPKERK pathways by binding miR-17/224 and miR-145, respectively [97,98].

Interestingly, many studies have indicated that circRNAs act as miRNA sponges regulating the expression of genes in plants [28,29]. For example, 854 circRNAs were identified in tomato, among which 163 differentially expressed (DE) circRNAs from fruits respond to cold stress, and 102 circRNAs serve as sponges that bind 24 miRNAs. For these miRNAs, multiple target genes were predicted to be involved in chilling processes, such as oxidation-reduction (redox) reactions, cell wall degradation, and heat and cold shock proteins [11].

#### 4.2.2. Predictive Web Tools for circRNA–miRNA Interactions

For a thorough study of the interaction between circRNAs and miRNAs in plants, this review draws on experiences from human life science research. Tools for predicting circRNA–miRNA interactions are comprehensively summarized. With scientific progress, tools for predicting the interactions of circRNAs and miRNAs in plants have been developed. We also provide a comprehensive introduction to bioinformatic tools, such as Circ2Traits, starBasev2.0, CircInteractome, and CircNet, and plant databases, such as AtCircDB, PlantCircNet, PlantcircBase, ASmiR, GreenCircRNA, and CircMiMi, which provide excellent platforms for predicting miRNA–circRNA interactions (Table 3) [99,100,101,102,103,104,105,106,107,108]. In addition, the “circRNA sponge” tool was the first pipeline developed to systematically identify circRNAs and their sponges from raw total RNA-seq and miRNA-seq files [109].

#### 4.2.3. Detection Methods for circRNA–miRNA Interactions

With advances in high-throughput sequencing technology and bioinformatics tools and methods, many circRNAs have been identified [24]. The central rationale for circRNAs acting as miRNA sponges is that circRNAs sequences bind miRNAs. Here, we describe strategies to validate interactions between circRNAs and miRNAs. Through these strategies, some targets of miRNAs and circRNAs can be used to further validate their biological functions.

(1) Luciferase reporter assays

Luciferase reporter assays are frequently used techniques to validate the associations of miRNAs with target miRNA response elements [71]. CircRNA fragments containing the target miRNA response elements and the mutant fragments are cloned into two different luciferase vectors, which are then transfected into cells in the presence of control or miRNA mimics. Finally, luciferase activity is measured with a reporter analysis system [71,110]. Luciferase reporter assays can be used to study the detailed binding sites of miRNAs and specific circRNAs.

(2) Antisense oligonucleotide (ASO) pulldown

CircRNAs’ unique backspliced junction sequences enable their detection and characterization. ASOs complementary to backsplice junctions are an important method for validating interactions between circRNAs and their target genes. After incubation with cell lysates, biotin-labeled circRNA-specific ASOs can be pulled down with streptavidin-coupled Dynabeads, and miRNAs associated with circRNAs can be identified through RT-qPCR and RNA-seq [72].

(3) Labeled microRNA pulldown assays

Labeled microRNA pulldown assays (LAMPs) are a simple and cost-effective in vitro technology that relies on the pulldown of miRNAs and their interacting target mRNAs [73]. LAMP has been successfully used to study the interactions of miRNAs with target mRNAs in zebrafish, as well as interactions of miRNAs and targeted long non-coding RNAs in plants. This technology has also been used for detecting miRNA-associated circRNAs [111].

(4) CircRNA–miRNA interaction assays

The novel circRNA–miRNA interaction (cmRRI) technology relies on biosensing systems. Bifunctional magnetic beads are used to capture circRNA–miRNA interactions and then amplify the signal to facilitate the detection of target molecules. Sensing magnetic beads containing DNA probes that recognize reverse splicing junction of circRNAs can be used to capture circRNAs and their interacting miRNAs [74].

(5) RNA fluorescence in situ hybridization

RNA fluorescence in situ hybridization (RNA-FISH) has been widely used to visualize the spatiotemporal localization of targeted RNAs in cells. RNA-FISH can be used to co-locate circRNAs and their target miRNAs [75]. The method relies on Cy3-labeled circRNAs and FAM or Alexa-labeled miRNAs, and hybridization with the two labeled RNAs, confocal microscopy imaging, and analysis of imaging results [112,113].

(6) Silencing and overexpression experiments

Modulation of circRNA levels by overexpression or silencing and analysis of effects on target mRNA abundance can be used to demonstrate miRNA sponge effects. Silencing or overexpression of target miRNAs or circRNA overexpression or silencing should reverse the effects on targeted genes in circRNA–miRNA–mRNA regulatory axes [76,77]. These methods have been widely used in studies of a variety of human diseases, and have shown that positive changes in circRNAs regulating target genes may be achieved through changing the bioavailability of miRNAs [77,114].

### 4.3. CircRNAs and Proteins

#### 4.3.1. CircRNA–Protein Interactions

The binding of circRNAs and proteins can be bidirectional; circRNA–protein interactions can regulate the synthesis and degradation of circRNAs, and also affect the expression and functions of proteins [115]. CircRNAs act as sponges or decoys of protein, thus affecting cellular functions and regulating gene transcription and the cell cycle [115]. CircRNAs interact with proteins, thus influencing the synthesis of circRNAs [52,115]. RBPs have been shown to regulate circRNA cyclization in various systems and organisms. RBPs include specific ADAR, QKI, FUS, nuclear factor NF90/NF110, DHX9, HnRNP, RBM20, MBL, SR, and epithelial splicing regulatory protein 1 (ESRP1). The interaction of circRNAs and proteins affects the degradation of circRNAs. In theory, the degradation of circRNAs may be initiated by endonuclease, followed by treatment with a combination of exonuclease and endonuclease. RNA modification via N6 methyladenosine (m6A) and poly (I:C) stimulation has been suggested to mediate the activation of endonuclide RNase L, and ultimately leads to the degradation of mRNAs and circRNAs [116,117].

In the medical field, circRNA–protein interactions have been found to affect protein expression and function. The effects on proteins during circRNA–protein interactions are divided into five main categories: (1) altering interactions between proteins; (2) binding or sequestering proteins, thereby preventing protein interactions with DNA, RNA, or other proteins; (3) recruiting proteins to chromatin, and binding of circRNAs to cis-acting elements and recruitment of transcription factors (TFs) or epigenetic modification enzymes that regulate gene expression; (4) formation of circRNA–protein–mRNA ternary complexes, and subsequent regulation of mRNA stability or direct regulation of protein translation; and (5) translocating or redistributing proteins [118]. Human disease studies have indicated interactions between circRNAs and proteins. For example, CUT-like homeobox 1 (CUX1)-generated circular RNA (circ-CUX1) enhances the trans-activation of MYC-associated zinc finger protein (MAZ) through EWS RNA-binding protein 1 (EWSR1), thereby promoting aerobic glycolysis and neuroblastoma progression [119]. CircFNDC3B (a circRNA originating from fibronectin type III domain-containing protein 3B), binds insulin-like growth factor II-binding protein 3 (IGF2BP3) and *CD44* mRNA, and forms a circFNDC3B-IGF2BP3-*CD44* mRNA ternary complex, then stabilizes mRNA and upregulates the expression of *CD44* [120]. In addition, circMYBL2 (derived from the cell cycle checkpoint gene *MYBL2*) facilitates protein translation and exacerbates acute myeloid leukemia by enhancing the interaction between polypyrimidine tract-binding protein 1 (PTBP1) and *FMS-like tyrosine kinase 3* (*FLT3*) mRNA [121]. However, relatively few studies have been reported in the field of botany.

#### 4.3.2. CircRNA–Protein Interactions Prediction Web Tool

The interactions between circRNAs and proteins are a major focus of current research. The development of tools for examining the interactions of circRNAs with proteins is extremely important. Many studies have examined the interactions between circRNAs and proteins in the fields of animal and human medicine, whereas research in plants has been relatively slower. In the fields of animal and human medicine, several predictive tools have been developed, for instance, catRAPIDomics v2 and CRMSS [122,123]. Many tools based on CLIP-seq as well as machine learning have been developed, for instance, CircSLNN, iCircRBP-DHN, RBPsuite, and CircRIP (Table 4) [124,125,126,127].

#### 4.3.3. Detection Methods for circRNA–Protein Interactions

On the basis of previous studies in the fields of animal life science, human medical science and plant science, the methods used to detect circRNA–protein interactions are summarized. These methods include RNase protection assay (RPA), RNA pulldown, RBP immunoprecipitation (RIP), electrophoretic mobility shift assay (EMSA), and fluorescence in situ hybridization (FISH) [115]. The details are as follows.

(1) RNase protection assays

The cyclic properties of candidate circRNA can be verified by treating total RNA with RNase R, which degrades linear RNA but not circRNA [128]. RPA assays can be used to map protein–RNA interactions. In these assays, RNase H is used to cut target RNA at a specific site hybridized with a DNA probe. When the protein binds the RNA at the target sequence, RNase H cleavage is prevented, thus indicating the sites of interaction between proteins and RNAs [52].

(2) RNA pulldown assays

In these assays, probes for binding known circRNAs are used for studying putative protein-binding chaperones. These probes are conjugated to streptavidin-coated magnetic beads. After the probes bind circRNAs, the binding of streptavidin-coated magnetic beads containing the targeted circRNAs and their binding protein complex are pulled down together. Subsequently, the beads are washed, and the bound proteins are confirmed by Western blotting or mass spectrometry [78].

(3) RNA immunoprecipitation

In contrast to RNA pulldown assays, RNA immunoprecipitation (RIP) detects RNA by targeting proteins of interest. The RIP method uses specific antibodies to proteins of interest to obtain targeted RNA from the complexes of RBPs and targeted RNA via immunoprecipitation. Thus, the RNAs associated with protein complexes are isolated and further analyzed through various methods, such as PCR, microarrays, Northern blotting, and sequencing analysis [79].

(4) Electrophoretic mobility shift assays

Electrophoretic mobility shift assay (EMSA) is an affinity electrophoresis technique frequently used to study protein–DNA and protein–RNA interactions [80]. The technique involves incubating a mixture of circRNAs and proteins, which are separated through polyacrylamide electrophoresis or agarose gel electrophoresis under natural conditions. At the end of electrophoresis, the circRNA species is determined by Western blotting with specific antibodies to the target protein [80].

(5) Fluorescence in situ hybridization and immunofluorescence

The colocalization of circRNAs and proteins through fluorescence microscopy has been used to identify circRNA–protein interactions. Among these technologies, FISH techniques can be used to detect circRNA–protein binding by detecting circRNA transcripts in cell or tissue samples via DNA oligonucleotide probes. Simultaneously, immunofluorescence staining can detect the location and abundance of proteins through specific fluorescently labeled antibodies [52]. Immunofluorescence technology involves steps of preparation, fixation, permeabilization, sealing, and use of fluorescent antibodies (or antigens) as probes to bind corresponding antigens (or antibodies) in tissues or cells and during incubation. Finally, tissue samples are examined through fluorescence microscopy [81]. The FISH method involves the fixation of cells or frozen tissue sections, permeabilization, fluorescence labeling, and hybridization of a target-specific probe consisting of 20 base pairs with the target RNAs. The hybridization signal amplification process can be performed at any step after hybridization washing. Finally, tissue samples are observed under fluorescence microscopy [82].

### 4.4. CircRNAs and mRNAs 

CircRNAs possess miRNAs binding sites, which bind specific miRNAs and form complexes, thereby inhibiting the binding of miRNAs and their target mRNAs and regulating target mRNA stability and transcription [129]. For example, circRNAMYLK directly binds miR-29a, thus increasing the expression of *vascular endothelial growth factor A* (*VEGFA*) mRNA and activating the VEGFA/VEGFR2 signaling pathway [130]. CircWDR77 inhibits the activity of miR-124, then upregulates the expression of miR-124-targeted *fibroblast growth factor 2* (*FGF2*) mRNA in vascular smooth muscle cells [131]. In addition, the *quinolinate synthase* (*QS*) mRNA-circQS-miR6024 regulatory network has been identified in tobacco. The *QS* gene and circQS are significantly upregulated after topping treatment, whereas miR6024 is downregulated [132]. In the *AO2* mRNA-circAO2-miRX282 regulatory network, the *AO2* gene is significantly upregulated after topping treatment, whereas circAO2 and miRX282 are slightly downregulated, thus suggesting that circRNAs may act as miRNA sponges and regulate expression of their parental genes [132]. During flag leaf development and senescence, circRNA 3:39797 208|39 848 986 acts as an osa-miR2925 sponge, thus regulating the expression of twenty-one target mRNAs. In addition, circRNA 8:5, 251, 329|5, 319, 040 was predicted as a possible sponge for osa-miR5074, thus regulating the expression of *Os07g0187900* mRNA [133].

In addition, circRNAs directly interact with mRNAs and regulate their translation [134]. CircRNA sequences may contain specific domains that interact with the 5′ or 3′ ends of mRNA, thereby affecting the translation efficiency of the mRNAs and the polypeptide. For example, biotinylated DNA probes targeting exon 2 of circZNF609 in cancer cells were used to capture the mRNAs interacting with circZNF609, and the interactions between circZNF609 and mRNAs were verified. The interaction of circZNF609 and *CKAP5* mRNA was found to enhance the stability and translation ability of *CKAP5* mRNA [135]. CircHomer1 is abundantly expressed in the orbitofrontal cortex in the brain and is derived from exons 2–5 of the HOMER homolog through backsplicing. CircHomer1 directly binds *Homer1b* mRNA (a synapse associated gene), and the binding site of *Homer1b* is located in its 3′ untranslated region. Because the predicted binding site is very close to the HuD binding element, the interaction of circHomer1-*Homer1b* mRNA decreases the expression of *Homer1b* mRNA [136].

CircRNAs also bind specific proteins and form complexes. These interactions may affect the structure and function of these proteins. In addition, circRNAs affect mRNAs translation by altering mRNA stability and post-transcriptional modification. CircRNAs therefore may alter the generation of proteins [137]. For example, *p21* (*cyclin dependent kinase inhibitor 1A*, *CDKN1A*) inhibits proliferation of HeLa cells. CircPCNX directly binds AU-rich element RNA-binding factor 1 (AUF1) and prevents AUF1 from interacting with *p21* mRNA, thereby increasing the abundance of *p21* mRNA. Therefore, circPCNX may enhance the expression of *p21* mRNA [138]. CircFAM120A, a common oncogenic circRNA, promotes the effective translation of *FAM120A* mRNA by preventing *FAM120A* mRNA from the binding of insulin-like growth factor 2 mRNA-binding protein 2 (IGF2BP2), thus promoting cell proliferation [139].

## 5. The Physiological Importance of circRNA in Plants

**Figure 3:**
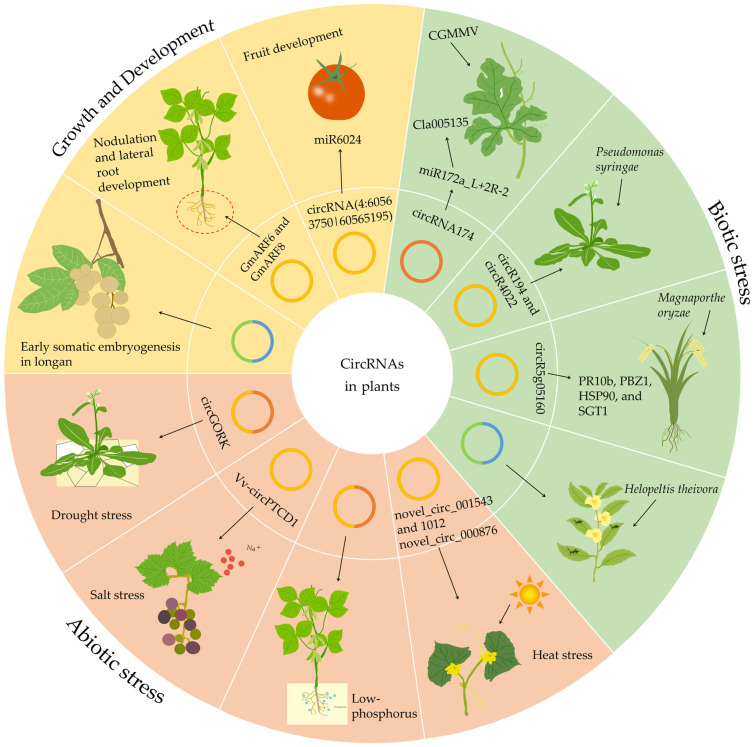
The significance of circRNAs in plants.

In humans and animals, circRNAs have been shown to participate in a wide range of various biological processes through various molecular mechanisms [140,141]. On the basis of research on circRNAs in humans and animals, the biological functions of circRNAs in plants have been analyzed. Several biological functions of circRNAs in plants are summarized below (Figure 3).

### 5.1. CircRNAs Serving as miRNA Sponges

To date, most research on plant circRNAs has remained preliminary, focusing on prediction of circRNAs as miRNA sponges, analyzing correlations between circRNAs and their parental genes, and exploring co-expression networks. Because of the abundant miRNA binding sites on transcripts, circRNAs specifically bind miRNAs and serve as competitive endogenous RNAs (ceRNAs), thereby sequestering miRNAs from their target mRNAs and negatively regulating miRNA function [29].

Potential regulatory networks of circRNA–miRNA–mRNA have been predicted [105]. A substantial proportion of circRNAs from various plant species have been identified as miRNA targets or miRNA sponges [14,18]. A prior study reported that 6.6% and 5% of circRNAs contain putative miRNAs binding sites in rice and *Arabidopsis*, respectively [21]. Another study identified 2354 circRNAs from rice, of which the 1356 are EcircRNAs. Among these 1356 EcircRNAs, 235 circRNAs contain miRNAs binding sites, and 31 circRNAs have two or more miRNAs binding sites [5]. According to the response of poplar (*Populus* L.) roots to various forms of nitrogen, thirty DEcircRNAs were found to have twenty-five corresponding miRNA binding sites, among which circRNA1010 has five different miRNAs binding sites [142]. One miRNA can bind multiple circRNAs. Moreover, one circRNA can bind multiple miRNAs [142]. Studies have indicated that the number of circRNAs with potential miRNA binding sites, as well as the number of potential miRNA binding sites within each circRNA, substantially varies across plant species [31].

Several studies have demonstrated that circRNAs play regulatory roles in various processes in plants, including metabolism, development, reproduction, and response to environmental stresses [25,42]. For instance, one study identified 176 DEcircRNAs between the early-flowering trifoliate orange (a mutant variant of *Poncirus trifoliata* L. Raf.) and the wild type; among them, 29 DEcircRNAs potentially bind sixteen miRNAs [143]. Additionally, three circRNAs were predicted to be targets of the miR172 family, and therefore may be involved in early flowering in trifoliate orange through the miR172 pathway [143]. In cotton, seven DEcircRNAs were found to bind seventeen miRNAs involved in the regulation of cotton fiber development [144]. Furthermore, in tomato, circRNA45 and circRNA47 were identified as potential miR477-3p sponges regulating the expression of their target *SpRLK1/2* mRNA, thus modulating *Phytophthora infestans* (*P. infestans*) [145]. Twenty-seven circRNAs were detected in melon (*Cucumis melo* L.), and found to potentially bind eighteen miRNAs or act as miRNA sponges. Some highly conserved miRNAs (such as miR156, miR159, miR167, and miR172) were predicted to be involved in disease resistance [146].

### 5.2. CircRNAs Regulate Gene Expression

In animals’ bodies, circRNAs form R-loop structures with parent genes via base pairing, and subsequently negatively regulate gene expression while increasing the expression of corresponding AS [70]. In recent years, research has increasingly examined circRNA-mediated regulation of gene transcription and expression in plants [25,31]. CircSEP3 is a nucleus-retained circRNA derived from exon 6 of *SEPALLATA3* (*SEP3*) in *A. thaliana*, which regulates the splicing of its linear counterpart. CircSEP3 strongly binds its homologous DNA sites and forms an R-loop, thus generating an alternatively spliced mRNA *SEP3* through exon jumping mode [147]. In moso bamboo, a significantly higher frequency of AS events was observed in genes producing circRNAs than randomly selected genes, thereby suggesting that circRNAs are involved in the AS process of these genes [50]. In maize, three types of circRNAs from the centromeric retrotransposon (CRM1) were found to promote chromatin looping by binding centromeres via R-loops. The looping of chromatin may influence transcription and the binding of chromatin regulatory factors [148]. In *Populus trichocarpa*, DNA–RNA hybrid immunoprecipitation (DRIP-seq) and sequencing revealed that the overexpression of circ-IRX7 in stem differentiating xylem increases the levels of R-loop structures, decreases expression of the parent gene *PtrIRX7*, and increases expression of the corresponding AS transcript *PtrIRX7-S* [149]. CircRNAs and their linear isoforms may inhibit the post-transcriptional expression of host genes through an overexpression genetic transformation system in rice [5]. Thus, the mechanism through which circRNAs regulate the expression of parent genes and homologous AS transcripts involves the formation of R-loop structures with genomic loci, thereby influencing the expression of parent genes and AS transcripts.

In addition, research increasingly indicates that circRNAs regulate the expression of their parental genes. For example, twenty-seven DEEcircRNAs were found to be induced under Pi-sufficient and Pi-starvation conditions in rice. Ten of eleven DE-parent genes were found to exhibit expression trends consistent with those of their corresponding circRNAs [21]. Slcirc108 and its parent gene, *Soly07g043420.2*, were found to be downregulated in tomato leaves after infection with tomato yellow leaf curl virus, whereas Slcirc017 and its parent gene, *Solyc01g080200.2*, are upregulated [150]. Overexpression of circRNAs affects the expression of parent genes. For instance, overexpression of *Os08circ16564* in rice decreases expression of its parent gene, *AK064900*, in transgenic plants [5]. In moso bamboo, protoplast transformation experiments showed that overexpression of circ-bHLH93 leads to decreased expression of its linear transcript, bHLH93 [50]. In tomato, overexpression of circ1-PSY1 derived from *phytoene synthase 1* (*PSY1*) inhibits the accumulation of its parent gene, *PSY1* [151].

### 5.3. Interactions between circRNAs and Proteins

In humans and animals, circRNAs interact with proteins [52,152]. The interaction of circRNAs and proteins may have bidirectional effects; circRNA–protein interactions influence the expression and function of proteins and also regulate the synthesis and degradation of circRNAs [115]. CircRNAs act as protein sponges or decoys that affect cellular functions, thereby regulating gene transcription and inhibiting the cell cycle [115]. Research on the interactions between proteins and circRNAs in plants is limited, but more than 1800 RNA-binding proteins have been identified in plants and found to play crucial roles in RNA splicing, editing, and transport [153]. These proteins are involved in regulating plant growth and development, as well as responding to various external stresses [154]. Some circRNAs have been identified from *Arabidopsis* Argonaute (AGO) immunoprecipitation libraries and therefore may affect miRNA function by interacting with AGO proteins [155]. CircRNAs have been discovered in *Arabidopsis* leaf extracellular vesicles, and found to bind glycine-rich RNA-binding protein 7 (GRP7) and sRNA-binding protein ARGONAUTE2 (AGO2); moreover, the proteins GRP7 and AGO2 contribute to the secretion and/or stabilization of these circRNAs [156].

### 5.4. CircRNAs Have Potential Translation Functions

In eukaryotes, translation always relies on the cap-dependent mechanism [157]. Generally, circRNAs are not involved in the translation to proteins because the closed structure of circRNA cannot bind ribosomal subunits [43]. However, circRNAs may not undergo cap-independent translation but may potentially rely on other translation factors, such as m6A and internal ribosome entry site (IRES) elements [28]. CircRNAs are rich in m6A modifications, which are sufficient to drive protein translation in a cap-independent manner, via the m6A reader YTHDF3 (a YTH N6-methyladenosine RNA-binding protein) and the translation initiation factors eIF4G2 and eIF3A [83]. Additionally, IRES elements form secondary structures on RNA, which can initiate translation in the absence or partial absence of classical translation initiation factors [84].

In plants, circRNAs have the potential to be translated into peptides or proteins [158,159]. In moso bamboo, forty-six circRNAs containing m6A modifications were identified through nanopore-based direct RNA-seq (DRS) technology, eleven of which may potentially be translated into long consecutive peptides [85]. In maize and *Arabidopsis*, 1463 ribosome-bound circRNAs were identified, 358 of which produce peptides [160]. At least one IRES element and an open reading frame were predicted on twelve circRNAs in soybean, which responds to low-temperature stress, thus suggesting that the circRNAs may potentially be translated into peptides or proteins [161]. A circRNA with an IRES is similar to a virus in rice and capable of initiating translation when the IRES element is inserted the upstream of the start codon AUG of the circRNA [86]. A total of 1569 circRNAs with potential translation activity were identified in *Arabidopsis* with CircCode software (https://github.com/PSSUN/CircCode accessed on 19 June 2024) [162]. By installing the specified program package and providing relevant data such as genome sequences and ribosome profiling data, potential translatable circRNAs can be identified [162]. However, research on m6A modifications and IRES elements in circRNAs in plants remains limited to date. Further in-depth studies to confirm the detailed translation processes are needed.

In summary, circRNAs in plants have several important functions, including serving as miRNA sponges, regulating transcription and splicing and thus controlling gene expression, participating in protein interactions, and being translated into peptides or proteins. Findings have revealed the crucial roles of circRNAs in plant gene regulatory networks and have provided new perspectives for deeper understanding of biological processes in plants. However, research on the functions and regulatory mechanisms of circRNAs in plants remains in early stages, and further in-depth studies are needed to elucidate the intricate regulatory networks.

## 6. Involvement of circRNAs in Plant Growth and Development

Plant development consists of three stages: embryogenesis, vegetative growth, and organogenesis development [163]. Plants are influenced by both external environmental factors and internal genetic regulation during their growth and development. Current research has focused primarily on the regulation of plant growth and development by protein-coding genes. With the development of high-throughput sequencing technologies, research on circRNAs in plants growth and development has attracted increasing attention. Most circRNAs are expressed in a tissue, organ, and developmental stage-specific manner in plants, thus forming a complex and intricate regulatory network [164].

CircRNAs often participate in the expression of genes involved in plant growth and development (Figure 3) [165]. For example, circRNAs primarily regulate pathways such as chlorophyll metabolism and hormone signal transduction, thereby regulating root, stem, and leaf growth; flower development; fruit ripening; and tissue senescence [49,166,167].

### 6.1. CircRNAs Involved in Plant Embryogenesis

The germination of seeds, which are involved in reproductive plant reproduction, is closely associated with the evolution of plant populations, and crop production and quality. Several studies have suggested circRNAs’ potential role in seed germination processes. For instance, a total of 347 DEcircRNAs were detected at 15 and 35 days after flowering between two peanut (*Arachis hypogaea* L.) varieties, named RIL 8106 (medium pod variety) and RIL 8107 (super pod variety). Annotation and enrichment analysis indicated that the DEcircRNAs are involved in the regulation of seed size and development [168]. A total of 3482 circRNAs were detected in sunflower seeds at different developmental stages, among which 316 DEcircRNAs are associated with fatty acid metabolism pathways and have regulatory roles in seed development, oil biosynthesis processes, and seed germination processes [169].

Furthermore, some circRNAs participate in the growth of plant seedlings. A total of 5029 circRNAs have been identified during three stages of early somatic embryogenesis (SE) in longan (*Dimocarpus longan* Lour.). Enrichment analysis using the Kyoto Encyclopedia of Genes and Genomes (KEGG) and Gene Ontology (GO) databases revealed the enrichment of the host genes of the DEcircRNAs in non-homologous end joining and succinic acid metabolism pathways [170]. These circRNAs may regulate the expression of homologous mRNA, thereby influencing the accumulation of related compounds and consequently affecting the development of early SE stages in plants [170]. In yellowing *Arabidopsis* seedlings, Ath_circ_476 and the parental gene *cytochrome C assembly protein* (*ycf5*) showed similarly significant downregulation trends, thereby indicating co-regulatory roles of circRNAs and their parental genes in affecting chloroplast accumulation and seedling growth [171].

### 6.2. Regulating Plant Nutritional Growth via circRNAs

Nutritional growth in plants refers to the differentiation and the formation of nutrient organs such as roots, stems, and leaves, from the germination of seeds or seedlings to the initiation of flower buds or young spikes. During this process, the meristematic tissues of roots and shoots play crucial roles. Roots are the primary organs for nutrient and water absorption in plants. Through root development and differentiation, plants efficiently absorb nutrients and water from the soil for the entire plant. CircRNAs play major roles in the nutritional growth process of plants by regulating gene expression and signal transduction through interacting with other bio-molecules, thereby influencing plant growth and development [172,173].

CircRNAs regulate root elongation in plants. A total of fifteen DEcircRNAs were identified in the root tips and shoot meristems of populus, and after overexpression of peu-miR160a, the expression level of one circRNA were downregulated. CircRNAs may bind peu-miR160a and subsequently participate in the maintenance and differentiation of root tips and shoot meristems in populus [172]. Ten DEcircRNAs were identified in wheat varieties YN29-LH9 and YN29-XN979. Because of the significantly larger and deeper roots of long-rooted plants (LH9 and XN979) than short-rooted plants (YN29), these DEcircRNAs are likely to be involved in regulating root length in wheat. Among them, three circRNAs may possess potential binding sites for miRNAs, and the expression levels of target miRNAs have shown significant differences between long-rooted and short-rooted plants [173].

Furthermore, circRNAs have the potential to regulate the formation of root nodules. The symbiotic nitrogen fixation of legume root nodules provides plants with a rich source of nitrogen. In soybean, *Auxin response factor 6* (*GmARF6*) and *Auxin response factor 8* (*GmARF8*) are associated with nodulation and lateral root development. Gm03circRNA1785 acts as a sponge for *GmARF6* and *GmARF8*, and regulates their expression [49]. A total of 3448 circRNAs identified in the roots of common bean (*Phaseolus vulgaris*) are specifically produced during symbiosis, and the highest numbers were found during the nitrogen fixation stage [174]. The circRNA–miRNA–mRNA regulatory network suggests that circRNAs may be involved in regulating transmembrane transport and kinase activity during nodulation and nitrogen fixation processes.

Beyond their potential to regulate root elongation and root nodule formation, circRNAs play roles in regulating the growth of shoots and leaves. CircRNAs may regulate the growth and development of plant leaves through various signaling pathways including hormone signaling pathways. CircRNAs are abundant in leaves, and show differential expression among leaf buds, young leaves, mature leaves, and senescent leaves. For instance, DEcircRNAs were identified between leaf buds and young leaves in tea plants, 54 of which have potential miRNA binding sites on target mRNAs. Cs-mi159-1, Cs-miR160-1, Cs-miR164a, and Cs-miR166a-1 were predicted to be targeted by more than five circRNAs. Homologous miRNA families may play roles in plant growth and leaf development. Therefore, circRNAs may have potential roles in the leaf development processes [17].

A total of 156 DEcircRNAs were found in winter dormant leaves and spring buds of tea plants, 22 of which interact with 346 miRNAs. Some circRNAs act as ceRNAs that interact with TFs, thereby regulating plant hormone biosynthesis and various signaling pathways in tea plants, and influencing cellular metabolism, growth, and development in tea leaves [175]. Six and thirty-five DEcircRNAs were detected during the growth-to-maturity and maturity-to-senescence stages in *Arabidopsis* leaves, respectively. CircRNAs may function during the senescence stage of *Arabidopsis* leaves by mediating porphyrin and chlorophyll metabolism, and hormone signaling pathways [167]. Additionally, 113 DEcircRNAs were identified in rice flag leaves from normal development to senescence. These findings indicate that senescence-associated circRNAs may participate in senescence processes in flag leaves through regulation of their parental genes and ceRNAs networks, primarily in oxidative-reduction processes, DNA damage repair, and hormone signaling [133].

### 6.3. Regulation of Reproductive Growth via circRNAs

#### 6.3.1. Regulation of Flower Development

Flowers, the reproductive structures of angiosperms, have substantial economic value. Flowering is a crucial physiological process in the life of higher plants, marking the transition from vegetative growth to reproductive growth. In recent years, studies have increasingly demonstrated that circRNAs play important regulatory roles in the development of plant flowers [176,177]. These circRNAs interact with other regulatory factors, thus influencing plant hormone signal transduction and regulating flowering pathways. For example, 4449 and 2209 DEcircRNAs were identified in female and male buds at undifferentiated, differentiated, and fully differentiated developmental stages in hickory (*Carya cathayensis* Sarg.), respectively. Functional annotation identified forty-six DEcircRNAs involved in flowering regulation [178].

The circRNA lariat41 is produced by the first intron of At5g37720 in *Arabidopsis*. Its overexpression leads to leaf curling, clustering, delayed flowering, and decreased fertility, thus indicating that lariat41 affects the development of leaves and flowers by regulating gene expression in *Arabidopsis* [179]. A total of 176 DEcircRNAs were found between early-maturing trifoliate oranges and the wild type, some of which regulate host genes encoding three zinc finger TFs, eight protein kinases, and three receptor-like kinases. Therefore, these circRNAs may be involved in early flowering of early-maturing trifoliate oranges [143].

Chr00:18744403|18744580-mdm-miR160 was predicted to play roles in flower formation and regulation of flower pigmentation in apple flowers [19]. Seventy-nine DEcircRNAs were detected at six developmental stages in *Rhododendron delavayi Franch* [180]. The overexpression of Rd-circR83 and Rd-circR103 significantly upregulates *Cytochrome P450 monooxygenase 704B 1* (*CYP704B1*) during the pollen stage. Furthermore, some mRNAs bound by DEcircRNAs are associated with development and aging processes, thus further suggesting circRNAs’ potential roles during the development and aging stages [180].

Pollen development plays a crucial role in plant fertility and genetics. CircRNAs were found to participate in pollen development. In *Brassica rapa*, 367 circRNAs were found to show stage-specific expression during pollen development. The parental genes of these circRNAs are involved primarily in pollen-associated molecular and biological processes, such as mitosis and meiosis [181]. A total of 165 DEcircRNAs were identified in anthers in Chinese cabbage (*Brassica campestris* L. ssp. *pekinensis*), among which 62 showed an upward trend, and 103 showed a downward trend. Seven circRNAs were suggested to be involved in the development of tapetum in Chinese cabbage. The DEmRNA of *BraA06g035480.3C* (*PPIase*), showing a trend toward upregulation, is regulated by circRNA105, which is crucial for microspore formation in Chinese cabbage [182].

Male sterile lines are important germplasm resources with essential roles in exploiting hybrid vigor in crops. CircRNAs are involved in the transformation of plant fertility. A total of 186 DEcircRNAs were identified in sterile rice lines compared with fertile rice varieties of Wuxiang. Of these, ninety-seven, eighty-seven, and sixty DEcircRNAs were identified during the stages of pollen mother cell (PMC) formation (P2), meiosis (P3), and microspore formation (P4), respectively [183]. The parental genes of DEcircRNAs are involved in biological processes such as development, stimulus response, hormone regulation, and reproduction. Additionally, fifteen of the DEcircRNAs are known putative miRNA sponges involved in the photo-thermal sensitive genic male sterile transformation of rice lines [183].

A total of 1009 DEcircRNAs were found between soybean cytoplasmic male sterile and maintainer or restorer lines. The parental genes of the DEcircRNAs are enriched primarily in metabolic processes, biological regulation, and reproductive processes, and many parental genes have been associated with flower development and male fertility [184]. In another study, a circRNA–miRNA–mRNA network was constructed in *Brassica campestris* L. ssp. *chinensis* Makino, and A02:23507399|23531438, and was predicted to be a circRNA involved in male sterility. By acting as an miRNA sponge, this circRNA regulates the expression of *Bra002750* by binding miRNAs unconservative_A06_21945 and unconservative_Scaffold000096_42992, thereby participating in the biosynthesis of the cuticle layer and the differentiation of pollen sperm cells [185]. CircRNAs not only affect the formation and development of flowers, but also are closely associated with pollen or anther development, and male reproductive ability. These findings may provide an important theoretical basis for hybrid breeding and reproduction.

#### 6.3.2. Regulation of Fruit Development

Fruit ripening, a crucial stage in fruit development, marks the period when fruit nutritional and flavor qualities gradually form. Ripening involves a complex genetic programming process including the regulation of fruit growth, coloration change, sugar accumulation, and synthesis of flavor compounds [186]. The regulatory mechanisms involve DEmRNAs’ numerous ripening-associated TFs [187,188].

CircRNAs regulate fruit development and ripening by interacting with miRNAs and RBPs, thereby regulating gene expression and influencing fruit ripening and quality [18,189]. A total of 282 DEcircRNAs were found between wild-type and transgenic *ethylene signaling transcription factor* (*LeERF*1) tomato fruits. In addition, sixty-one circRNAs were found to be potential ceRNAs binding forty-seven miRNAs, among which miR6024 is a target of circRNA (4:60563750|60565195). Target prediction indicated that miR6024 is involved in ethylene biosynthesis and signal transduction pathways. Therefore, circRNA may play important roles in the ethylene pathway [190].

A total of 340 DEcircRNAs were detected in ripe green and red tomato fruits, and 19 DEcircRNAs were found to act as miRNA sponges regulating the expression of 94 target mRNAs. Among these miRNAs, miR5303 is targeted by fourteen circRNAs, and forty-two mRNAs are targeted by miR5303, thus suggesting the importance of miR5303 in fruit ripening [189]. A total of 252 DEcircRNAs were identified in sea buckthorn fruit development, and the host genes of these DEcircRNAs were predicted to be involved in carotenoid biosynthesis, lipid synthesis, and plant hormone signal transduction. Additionally, fifty-three DEcircRNAs were predicted to act as sponges for nine miRNAs, thus revealing the roles of circRNAs in sea buckthorn fruit ripening [18]. A total of 238 circRNAs were found in two stages of cucumber fruit development (at 5 and 10 days after pollination), of which 51 are DEcircRNAs. Multiple miRNAs target multiple circRNAs, and circRNAs regulate fruit ripening genes including *WRKY domain transcription factors*, *MADS box proteins*, and *shedding stress maturation inhibitors* [191].

The accumulation and degradation of pigments are crucial during fruit coloration and ripening, and directly lead to changes in fruit color. Multiple studies have indicated that circRNAs participate in regulating fruit quality characteristics such as color. Compared with tomato fruits in the mature green stage, significant changes in the expression levels of 273 and 89 circRNAs were identified in fruits at the ripening stage for two varieties of Jinling Fenyu and Jinling Moyu, respectively. According to GO enrichment analysis, the parental genes of DEcircRNAs are involved primarily in metabolism, cellular and single-organism processes, and have roles in catalytic activity and binding, thus suggesting that circRNAs are involved in regulating fruit coloration and ripening [192]. The overexpression of circRNAs originating from the genes *phytoene synthase 1* (*PSY1*) and *phytoene desaturase* (*PDS*) in tomatoes decreases the accumulation of lycopene and *β*-carotene in tomato fruits, as well as the expression of their parental genes and corresponding pigment content [151].

## 7. Roles of circRNAs in Plant Stress Responses

Plants may encounter various biotic and abiotic stresses throughout their lifecycle. Biotic stresses arise predominantly from attacks by pathogens, pests, and weeds, whereas abiotic stresses encompass factors such as extreme temperatures, drought, salinity, nutrient imbalance, and light intensity negatively affecting plant growth and development [193]. Whether plant circRNAs play roles in the stress response is a direction worthy of attention.

Many studies have suggested that circRNAs may have roles in the responses of plants to both biotic and abiotic stresses (Figure 3) [165,194,195]. In those studies, numerous DEcircRNAs have been identified and found to exhibit distinct expression patterns under various stress conditions (Table 5).

### 7.1. Responses of circRNAs to Biostress

Plants are susceptible to a multitude of threats including pathogens (viruses, bacteria, and fungi), pests, and weeds, which cause severe damage to plant growth and substantially threaten agricultural productivity. In recent years, the roles of circRNAs in defense of plant diseases and pests have attracted increasing research attention. CircRNAs have been found to play important roles in plant diseases resistance, interactions between pathogens and hosts, signal transduction, and hormone regulation [196]. Some circRNAs are involved in regulating extracellular, intracellular, and intercellular signaling pathways, thereby modulating the interactions between plants and pathogens or pests, as well as signal transduction in disease resistance and the host defense response.

#### 7.1.1. CircRNAs and Plant Viral Infections

CircRNAs from plants exhibit differential expression in response to viral infection. CircRNAs from plant hosts participate in viral resistance by regulating the expression of miRNAs or target genes. A total of one hundred fifty-five and five DEcircRNAs were found to be significantly upregulated and downregulated, respectively, in maize leaf after infection with maize Iranian mosaic virus. These findings indicate a circRNA expression response to viral infection in plant host. Additionally, thirty-three circRNAs were predicted to bind twenty-three miRNAs and to potentially affect plant metabolism and development, thus suggesting roles of circRNAs in regulating defense responses to viral infection [197]. Eighty-three and thirty-two DEcircRNAs were found in tomato leaves with and without infection with tomato yellow leaf curl virus (TYLCV). *Solyc07g043420.2.1* is the parental gene of Slcirc107. TRV-Solyc07g043420.2.1 was transiently silenced through a virus induced gene silencing technique, and the parental gene of Slcirc107 was found to be involved in TYLCV infection [150]. A total of 548 DEcircRNAs were identified in watermelon leaves infected with cucumber green mottle mosaic virus (CGMMV), among which 153 are sponges for 88 miRNAs [13]. CircRNA174 has been suggested to act as a ceRNA, serving as a decoy for miR172a_L+2R-2 during CGMMV infection, and to regulate expression of the *Cla005135* gene from the MYB family. Additionally, circRNA214 might regulate the expression of *cytochrome P450*, *hexokinase*, *WRKY*, and *lipoxygenase* genes by competing with miR6300 as a competitive endogenous target mimics (eTMs) [13]. A total of 107 and 58 DEcircRNAs were found in tomato leaves infected with tomato leaf curl Bangalore virus and uninfected leaves, respectively, among which 103 DEcircRNAs are exonic circRNAs regulating the expression of their parent genes. A significant positive correlation was also identified between the expression patterns of circRNAs and their parental genes during different intervals of viral infection, thereby indicating the involvement of circRNA-mediated gene expression in viral resistance [198].

#### 7.1.2. CircRNAs and Bacterial Infections

Plant bacterial diseases lead to plant death, or significantly decreased crop yield and quality. Increasing evidence suggests that circRNAs exhibit differential expression patterns in plants during bacterial infection. CircRNAs function as miRNA sponges, or interact with proteins, and regulate the expression of genes associated with disease resistance or pathogenicity, then inhibit or decrease the occurrence of bacterial diseases. For instance, a total of 429 DEcircRNAs were detected in the potato varieties Valor (susceptible) and BP1 (disease tolerant) infected with *Pectobacterium carotovorum* subsp. *brasiliense* (*Pcb*) in carrot soft rot. These DEcircRNAs participate in defense responses, cell wall integrity, and phosphorylation pathways, thus highlighting their roles in regulating potato immune responses [10]. A total of 584 DEcircRNAs in kiwifruit were found to respond to infection by *Pseudomonas syringae* pv. *actinidiae* (Psa), the causative agent of bacterial canker disease. A subset of circRNAs closely associated with plant defense responses play critical roles in plant immune reactions [16]. CircR194 and circR4022 from *Arabidopsis* are involved in resistance responses to *Pseudomonas syringae* infection in *A. thaliana*, and circR11208 enhances host resistance to *Botrytis cinerea* infection [199]. A total of 276 DEcircRNAs were identified in rice leaves infected with *Xanthomonas oryzae* pv. *oryzae* (*Xoo.*). Among them, 31 DEcircRNAs have predicted binding sites for 121 miRNAs. These DEcircRNAs may play important roles in the defense response of rice against pathogens during *Xoo.* infection by mediating photorespiration, as well as chloroplast, peroxisome, and diterpene biosynthesis [200]. In another study, fourteen DEcircRNAs were identified from rice leaf infected with *Xoo.*, among which ciR52, ciR298, and ciR133 are induced by *Xoo.* infection. CiR133 suppresses the expression of its parent gene, *OsARAB* (putative *arabinofuranosidase* gene), during *Xoo.* infection. Overexpression of ciR133 and *OsARA* mutation was found to enhance rice resistance to *Xoo.* infection [201].

#### 7.1.3. CircRNAs and Fungal Infections

Most plant diseases are caused by fungal infections, which often decrease crop quality and lead to yield loss. CircRNAs have been extensively studied in fungal infected plants. Moreover, circRNAs regulate the expression of target genes by acting as miRNA sponges or by directly modulating the expression of genes associated with the defense response in plant hosts and pathogenic process. A total of 636 DEcircRNAs were identified in rice infected with *Magnaporthe oryzae*. Overexpression of circR5g05160 upregulates the expression of *PR10b*, *PBZ1*, *HSP90*, and *SGT1* genes, and enhances rice resistance to the pathogen [202]. After infection with *P. infestans*, compared with control plants, transgenic tomato overexpressing circRNA45 and circRNA47 was found to exhibit smaller lesion areas and lower expression of miR477-3p. Therefore, circRNA45 and circRNA47 may positively regulate tomato resistance by modulating miRNA–mRNA expression levels [145]. A total of seventeen and twenty-three DEcircRNAs were identified in two melon varieties, B29 (powdery mildew-sensitive variety) and M1 (powdery mildew-resistant variety), after infection with powdery mildew. On the basis of the functional annotation of parent genes of circRNAs, circRNAs may participate in responses to biological stimuli, redox reactions, metabolic processes, and regulation of gene expression. Additionally, twenty-seven circRNAs were predicted to be potential targets or sponges of eighteen miRNAs, thus suggesting that circRNAs may regulate gene expression leading to resistance against powdery mildew infection in melons [146]. After infection with Verticillium wilt in cotton, 280 DEcircRNAs were identified, and the number of DEcircRNAs in susceptible genotypes was approximately twice that in resistant genotypes. Among the parental genes of seventeen DEcircRNAs, thirteen genes were annotated into non-redundant genes, and eleven of thirteen parental genes were found to be *nucleotide binding site* (*NBS*) family genes [203]. *NBS* genes may play roles in cotton resistance to Verticillium wilt through regulation by their circRNAs [203]. In ginseng (*Panax ginseng* Mayer), after it was infected with ginseng rusty root, sixteen DEcircRNAs involved in fatty acid-associated regulation were identified. Alterations in fatty acid-associated pathways are believed to play crucial roles in these symptoms [204]. In soybean infected with wilt disease, twenty-four DEcircRNAs were detected, eighteen of which act as endogenous target mimics for ten miRNAs, thus potentially regulating genes involved in wilt disease stress tolerance [205].

In *Brassica rapa* inoculated with *Plasmodiophora brassicae*, 231 DEcircRNAs were identified. The upregulated novel_circ_000495 was found to suppress the expression of miR5656-y, thus upregulating the target gene *Bra026508* and potentially altering plant resistance. A study of host genes of circRNAs suggested that circRNAs are involved primarily in the biosynthesis of terpenes, flavonoids, and alkaloids [206]. Tea leaf spots caused by *Lasiodiplodia theobromae*, *Didymella segeticola*, *Didymella bellidis*, *Epicoccum sorghinum*, *Epicoccum nigrum*, *Epicoccum mackenziei*, *Pestalotiopsis trachicarpicola*, or *Alternaria longipes* affect the production and quality of tea leaves in Southwest China [207,208,209,210,211,212,213,214]. A total of 192 and 153 DEcircRNAs were found to be significantly upregulated and downregulated in tea leaf spots caused by *Lasiodiplodia theobromae* [215]. Seventeen DEcircRNAs were predicted to bind twenty-nine miRNAs in tea leaf spots caused by *D. segeticola*, and functional annotation analysis indicated that the host genes of these circRNAs may play important roles in pathways involved in plant–pathogen interactions [216]. The interactions between individual ceRNAs and miRNAs were predicted in tea leaf spots caused by *P. trachicarpicola*, and some interactions in the circRNA–miRNA–mRNA network in infected tea leaves were found ultimately to alter mRNA abundance [217].

#### 7.1.4. CircRNAs and Insect Attacks

Limited research has indicated circRNAs’ regulatory roles in response to insect attacks in plants. Some studies have demonstrated the involvement of plant circRNAs in response to pest attacks. For example, 199 DEcircRNAs were identified between resistant and susceptible soybean under cotton bollworm (*Helicoverpa armigera*) damage. Functional annotation analyses revealed that the host genes from these DEcircRNAs associated with various biological processes are activated during chewing damage caused by pests. Additionally, 1118 circRNAs in soybean were estimated to have abundant miRNA binding sites. Some host genes of circRNAs were predicted to be involved in insect resistance processes, such as pyridine and piperidine alkaloid biosynthesis, as well as monoterpenoid biosynthesis [9]. Thirty-four circRNAs from tea plants were found to be up or downregulated in response to *Helopeltis theivora* attack. Functional annotation and enrichment analyses revealed that the target genes of circRNAs are enriched in biological pathways such as tryptophan metabolism and porphyrin metabolism. Additionally, seventeen DEcircRNAs were found to act as miRNA sponges, thereby preventing miRNA–mRNA binding and inhibiting mRNA degradation [218].

In conclusion, research on circRNAs in biological stress is important for understanding plant responses to biostress, such as pathogenic infection; enhancing crop resistance to diseases and pests; and promoting the sustainability of agriculture. Further understanding of the functions and regulatory mechanisms of circRNAs would provide new insights and strategies for crop improvement, disease control, and sustainable agricultural development. However, further experiments and studies remain needed to elucidate the specific functions of circRNAs and their regulatory mechanisms in biological stress, as well as their effectiveness and potential practical applications.

### 7.2. CircRNA Response to Abiotic Stresses

Plant growth is highly susceptible to external non-biological stresses, such as temperature stress (heat stress, freezing, and chilling), water stress (drought and flooding), salt stress, and nutrient stress (calcium, nitrogen, phosphorus, and potassium) [219]. Increasing evidence suggests that the expression of circRNAs changes when plants are subjected to abiotic stresses, thereby influencing plant metabolism, physiology, and growth. However, the biological importance of circRNAs and their regulatory functions under these conditions remains to be elucidated.

#### 7.2.1. CircRNAs and Temperature Stress

Temperature stress is the most important abiotic stress to which plants are subjected. This stress includes both heat and cold stress, which disrupt normal plant growth and development. CircRNAs from plants have been shown to react to changes under lower and higher temperatures [220,221]. Under heat stress, the number of circRNAs was found to significantly increase from 488 to 1583 in *A. thaliana*, and 439 DEcircRNAs were identified, some of which are positively correlated with their respective host genes. Moreover, heat-induced circRNAs may potentially engage in plant responses to heat stress through circRNA-mediated ceRNA networks [222].

In cucumbers subjected to heat stress, five and one circRNA were found to show upregulation and downregulation trends, respectively. In addition, novel_circ_001543 and novel_circ_000876 were identified as potential regulators of the plant hormone signaling pathway by serving as miR9748 sponges and targeting relevant genes [223]. Germination of tomato seeds under high-temperature conditions, seventy-three DEcircRNAs have been found to show upregulation- or downregulation trends. GO annotation analysis revealed that the host genes of circRNAs in these seeds are involved primarily in metabolic processes, cellular processes, catalytic activity, and binding processes. These findings indicated the responsiveness of certain circRNAs from seeds under heat stress during the germination phase [224]. Beyond heat stress, extreme cold also damages plants [225]. Several studies have indicated that circRNAs are differentially expressed under low temperature stress [220,221]. In tomato fruits, 163 circRNAs were found to respond to cold stress, and 862 target genes of circRNAs were found to be associated with cold damage, such as redox reactions and crucial enzymes for cell wall degradation [11]. In tomato leaves, 1759 DEcircRNAs were found to be induced by low-temperature treatment, and 383 DEcircRNAs were found to serve as sponges for 266 miRNAs, thus collectively targeting 4476 mRNAs. These circRNAs may participate in regulating pathways associated with metabolism and signal transduction [226].

Additionally, a comparison of control treatment and freezing treatment in bell pepper identified thirty-six DEcircRNAs: twenty-eight upregulated and eight downregulated [12]. The overexpression of Vv-circATS1 (a circRNA derived from the parental gene *glycerol-3-phosphate acyltransferase*) in transgenic plants significantly enhances cold resistance in *A. thaliana* [48]. Moreover, mRNA-seq data analysis indicated that Vv-circATS1 regulates the expression of miRNAs and various stress-responsive genes, thus modulating the response to cold stress in grapevines. These genes include *Copper/zinc superoxide dismutase 2* (*CSD2*), *peroxidase CA* (*PRXCA*), *pectin methylesterase 41* (*PME41*), *Lipoxygenase 3* (*LOX3*), and *WRKY48*, which are upregulated with overexpression of Vv-circATS1 in *A. thaliana* [48]. In the ceRNA regulatory network, Gma_circ_0000098 was found to bind miR1533, whereas miR1533 was found to bind multiple genes involved in the low-temperature response, including *serine/threonine protein PP2A*, *polygalacturonase*, *Basic Leucine Zipper* (*bZIP*), and *lysine-specific demethylase 1* (*LSD1*). CircRNAs acting as miRNA sponges may play crucial roles through affecting target mRNAs that enhance low-temperature tolerance in plant hosts [161]. In tea plants, 250 DEcircRNAs were identified under cold stress, and a key circRNA, Cs-circFAB1, was verified to be involved in cold tolerance in tea plants through FISH and silencing experiments [227].

#### 7.2.2. CircRNAs and Water Stress

Water, a crucial component of plant physiology and biochemistry, is essential for maintaining intrinsic morphology. Water stress comprises primarily drought and flooding. Drought is a major abiotic stress detrimental to plant growth and development in most regions [228]. Numerous studies have confirmed the major roles of circRNAs in plants’ responses to drought stress [6,229]. Some circRNAs regulate ion balance and water regulation in plants, thereby enhancing their tolerance to drought stress [28]. For example, sixty-two DEcircRNAs were identified in dehydrated wheat seedlings, six of which act as sponges for twenty-six miRNAs. These circRNAs participate in dehydration response processes such as photosynthesis, porphyrin, and chlorophyll metabolism [6].

In *Arabidopsis*, the overexpression of circGORK (from Guard cell outward-rectifying K^+^-channel) is associated with a relatively lower water loss rate and higher survival rate under drought conditions, thus indicating that circGORK is a positive regulator of the response to drought stress in transgenic plants [229]. Thirty-three DEcircRNAs were identified in birch-leaf pear and found to be involved in various dehydration response processes, such as metabolic pathways and amino acid biosynthesis. Moreover, 309 circRNAs were predicted to act as sponges for 180 miRNAs, thereby indicating their involvement in the drought response process [20]. Fifty-two DEcircRNAs were identified in moso bamboo under drought conditions, twenty of which have miRNA binding sites. These DEcircRNAs participate in biochemical processes in response to drought, such as amino acid biosynthesis, and plant hormone signal transduction, including that of abscisic acid [230]. The circRNA–miRNA–mRNA network constructed in sugar beet (*Beta vulgaris* L.) revealed that novel_circ_0000442 and novel_circ_0000443 may sponge Ath-miR157d and regulate the expression of its target genes involved in drought response, including *BVRB_1g004570*, *BVRB_1g005450*, and *BVRB_1g005790*. Some parent genes of drought-responsive circRNAs are associated with signal transduction and oxidative-reduction processes [231].

#### 7.2.3. CircRNAs and Salt Stress

Excessive soluble salts in soil often lead to plant osmotic stress or ion stress [232]. Salt stress inhibits the growth and development of plant roots [233]. CircRNAs respond to salt stress in plants. For example, when cucumber is subjected to salt stress, 1934 and 44 DEcircRNAs were identified in roots and leaves, respectively. Several parental genes of DEcircRNAs in roots were identified as *serine/threonine protein kinases* genes, including *SNF1-related kinases* (*SnRKs*) and *PP2C* [14]. As members of signal transduction pathways, serine/threonine protein kinases are involved in tolerance to various stresses in plants, including salt, drought, freezing stress and senescence [234,235,236].

A total of 107 DEcircRNAs were identified in the roots of two tomato varieties under salt stress; sly_circ_2651 may potentially enhance the expression levels of *laccase enzyme* (*LAC*) mRNA by interacting with sly-miR397-5p, thereby increasing salt tolerance [237]. Moreover, ninety-three and ninety-five DEcircRNAs were found in the rice roots of the salt-susceptible recipient cultivar (93-11) and the salt-tolerant introgression line (9L136) under salt stress, and twenty-seven and thirty-seven miRNAs were found to competitively bind nineteen and twenty-six DEcircRNAs in 9L136 and 93-11 rice varieties, respectively. These circRNAs play roles in regulating physiological and biochemical processes, including transcription, signal transduction, translation, secondary metabolism, and inorganic ion transport [238]. In rice, *Os02circ25329*, *Os06circ02797*, *Os03circ00204*, and *Os05circ02465* genes were knocked out through the CRISPR-Cas9 technique. The deletion rate of these genes in protoplasts and stable transgenic T0 lines was found to exceed 10%. Phenotypic identification showed that these circRNAs are involved in the salt stress response during seed germination. Notably, the *Os05circ02465* deletion mutant shows high salt tolerance [239]. Overexpression of Vv-circPTCD1 (whose parental gene is *pentatricopeptide repeat domain-containing protein 1*, *PTCD1*, in grape), in *A. thaliana* seedlings, had a lower survival rate on NaCl-containing medium than the WT, and most seedlings died after salt stress. These results also suggest that Vv-circPTCD1 may act as a negative regulator of salt stress [240].

#### 7.2.4. CircRNAs and Nutrient Stress

Nutrient stress is a common environmental stress for plants. Plant growth and development require a large stable supply of essential nutrients, including nitrogen, phosphorus, potassium, calcium, and magnesium [241]. In the presence of an insufficient or excessive supply of nutrients in the environment, plant hosts often trigger a series of responses involving cellular and molecular level regulation [242]. CircRNAs regulate miRNA function and participate in plant responses to nutrient stress [7,237]. Nitrogen and phosphorus are essential nutrients for plant growth and development. Insufficient or excessive levels of nitrogen and phosphorus affect various aspects of the plant lifecycle. Multiple studies have identified DEcircRNAs under nitrogen and phosphorus stress. For example, in wheat, six and twenty-three DEcircRNAs were found to be involved in the host response to low nitrogen (LN) stress or regulating root growth under LN conditions, respectively. Additionally, seven DEcircRNAs were found to act as miRNA sponges, thus suggesting their important roles in plant responses to low nitrogen conditions [243]. Furthermore, twenty-four and twenty-two DEcircRNAs were identified in maize leaves and roots under high nitrogen and LN conditions, respectively. Thirty-four circRNAs were found to act as miRNA sponges, thus potentially playing important roles in biological processes such as biosynthesis and the metabolism of organic nitrogen compounds [244]. In poplar roots, thirty DEcircRNAs were found to act as miRNA sponges for twenty-five miRNAs, and some DEcircRNAs specifically target miR169 and miR396—well-known miRNA family members with key roles in the nitrogen response and plant growth and development [142]. A total of 120 DEcircRNAs were identified in soybean under low phosphorus stress, and the expression of circRNAs in the LP-tolerant genotype has been found to be more stable than that in the LP-sensitive genotype. The parental genes of circRNAs and targeted genes of miRNAs were found to be enriched in defense responses, ADP binding, oxidative enzyme activity, and signal transduction processes, according to GO annotation [245]. In addition, twenty-seven DEcircRNAs were identified in rice under of phosphorus deficiency, thus suggesting their potential roles in response to rice Pi starvation stress [21].

Furthermore, circRNAs are responsive to calcium deficiency and copper toxicity [246,247]. Twenty-three and twenty-two DEcircRNAs were found in Chinese cabbage in various stages of calcium deficiency, compared with controls, and these DEcircRNAs may participate in processes such as stimulus response, ATP enzyme activity, and cell wall metabolism. CircRNAs are involved in plant tip damage induced by calcium deficiency, through diverse biological processes and physiological functions [246]. Additionally, forty-five and seventeen DEcircRNAs were identified in the roots and leaves of Ziyang Xiangcheng (*Citrus junos* Sieb. Ex Tanaka) under excess copper, respectively. These circRNAs may function as ceRNAs that sequester copper-responsive miRNAs and consequently inhibit their function [247].

**Table 5:** CircRNAs in plants stress responses.

Types of Stress to Plants	Stimuli in the Environment	Plant Species	Tissues	Number of Differentially Expressed circRNAs	Reference
Viral infections	Maize Iranian mosaic virus	Maize	Leaf	160	[197]
	Tomato yellow leaf curl virus	Tomato	Leaf	115	[150]
	Cucumber green mottle mosaic virus	Watermelon	Leaf	548	[13]
	Tomato leaf curl Bangalore virus	Tomato	Leaf	165	[198]
Bacterial infections	*Pectobacterium carotovorum* subsp. *brasiliense*	Potato	Stem	429	[10]
	*Pseudomonas* syringae pv. *actinidiae*	Kiwifruit	Leaf	584	[16]
	*Xanthomonas oryzae* pv. *oryzae*	Rice	Leaf	276	[200]
Fungal infections	*Magnaporthe oryzae*	Rice	Leaf	636	[202]
	*Powdery mildew*	Melon	Leaf	40	[146]
	*Verticillium wilt*	Gossypium	Root/stem	280	[203]
	Wilt disease	Soybean	Leaf	24	[205]
	*Plasmodiophora brassicae*	*Brassica rapa*	Root	231	[206]
	*Lasiodiplodia theobromae*	Tea	Leaf	345	[215]
Insect attacks	Cotton bollworm	Soybean	Leaf	199	[9]
	*Helopeltis theivora*	Tea	Leaf	34	[218]
Temperature stress	Heat stress	*Arabidopsis*	Seedling	439	[222]
	Heat stress	Cucumbers	Leaf	6	[223]
	Heat stress	Tomato	Seed	73	[224]
	Low-temperature treatment	Tomato	Leaf	1759	[226]
	Chilling	Bell pepper	Fruit pericarp	36	[12]
	Cold stress	Tea	Tender bud or young leaves	250	[227]
Water stress	Dehydration Stress	Wheat	Seedling	62	[6]
	Drought stress	Birch-leaf pear	Leaf	33	[20]
	Drought Stress	Moso bamboo	Leaf	52	[230]
	Drought Responses	Sugar beet	Leaf	17	[231]
Salt stress	Salt stress	Cucumber	Root/leaf	1934/44	[14]
	Salt stress	Tomato	Root	107	[237]
	Salt stress	Rice	Root	188	[238]
Nutrient stress	Low nitrogen stress	Wheat	Root	29	[243]
	Nitrogen stress	Maize	Leaf/root	24/22	[244]
	Low phosphorus stress	Soybean	Root	120	[245]
	Phosphorus deficiency	Rice	Root	27	[21]
	Calcium deficiency	Chinese cabbage	Leaf	23	[246]
	Excess copper	*Citrus junos Sieb*. Ex Tanaka	Root/leaf	45/17	[247]

The mechanisms through which circRNAs regulate plant growth and development, or responses to environmental stress, are not fully understood. However, considerable evidence indicates that these RNAs regulate plants growth and development, and respond to biotic and abiotic stresses by acting as miRNA sponges, thereby regulating miRNAs and target genes. In addition, circRNAs regulate plant hormone signaling pathways, such as ethylene, abscisic acid, and jasmonic acid pathways [190,229,248]. Additionally, circRNAs in plants may regulate specific biological processes; examples include circRNAs involved in nicotine synthesis in tobacco (*Nicotiana tabacum*), secondary metabolite biosynthesis in *Salvia miltiorrhiza*, and starch and sucrose biosynthesis in taro (*Colocasia esculenta*) [132,249,250]. These studies have expanded our understanding of the roles of circRNAs in plant growth, development, and responses to biotic and abiotic stresses.

## 8. Conclusions and Future Prospects

CircRNAs, a class of non-coding RNAs, have diverse biological functions in plants. Over the past several years, circRNAs have emerged as a focal point of research in plants. Many studies have shown that circRNAs are widely expressed in a variety of plant species and exhibit spatiotemporal tissue-specific expression patterns. Additionally, many circRNAs are generated only in specific developmental stages or specific tissues in plants, or under particular stress conditions. Therefore, cutting-edge bioinformatics techniques such as high-throughput sequencing and machine learning will enable more efficient prediction and identification of circRNAs. High-throughput sequencing and machine learning can be leveraged to more effectively predict and identify additional circRNAs.

CircRNAs exert their biological functions primarily by serving as miRNA sponges, regulating gene transcription and the expression of their parental genes, interacting with proteins, and being translated into peptides or proteins. In plants, circRNAs are involved in multiple critical biological processes such as growth and development, and responses to biotic and abiotic stresses. For example, circRNAs in plants induce adaptation to salt stress, promote root development, and enhance plant immunity, among other functions. Furthermore, circRNAs interact with other bio-molecules and form RNA–RNA complexes, which further regulate gene expression. Through these modes, circRNAs function similarly to TFs and microRNAs as regulatory factors in growth and development processes of plants.

In summary, the findings have revealed not only the complexity of the circRNA regulatory networks in plants but also important clues for the early detection of plant diseases. Moreover, correlation analysis between the expression of plant circRNAs and their parental genes provides new avenues for investigation. By using comprehensive databases and bioinformatics tools, further advancements in circRNA functional studies can be achieved. Developing plant-specific circRNA analysis software and comprehensive plant databases will enhance the accuracy of prediction and analysis.

Despite substantial progress in understanding the functions of circRNAs in plants, many potential directions remain for further exploration in this field. Future research may focus on the following aspects:

(1) Functional validation: Although circRNAs in plants are known to exhibit diverse biological functions, many conclusions have been based on RNA-seq analysis and bioinformatics prediction. The biological functions of circRNAs are often inferred through GO or KEGG annotation and enrichment analyses of their parental genes, whereas few studies have validated the biological functions of circRNAs. Additionally, challenges exist in the technologies for overexpressing or silencing circRNAs in plants, such as avoiding effects on the expression of parental genes when knocking down or knocking out circRNAs, or minimizing interference from exogenous genes and neighboring genes.
(2) In-depth investigation of the regulatory mechanisms of circRNAs: The mechanisms underlying circRNA biogenesis and degradation, as well the study of circRNA–miRNA interactions and regulation of circRNAs’ downstream genes, should be explored. These studies would further elucidate the biological functions and regulatory mechanisms of circRNAs, and aid in understanding the functions of circRNAs in processes such as plant growth, development, and aging.
(3) Revealing the interactions between circRNAs and other biomolecules (such as miRNAs and proteins): Beyond circRNAs, many other biomolecules are present in plants, such as miRNAs, long non-coding RNAs, and proteins, some of which may have functional crosstalk or interactions with circRNAs. Future research should investigate the interactions among these biomolecules, and elucidate their synergistic roles in regulating gene expression in plants. This exploration would reveal additional details regarding their involvement in plant growth, development, and stress responses.
(4) Application of circRNAs as biomarkers: In plants, circRNAs serve as biomarkers for AS variants of plant exons, and the potential applications of biomarkers in plant breeding have been explored. Thorough understanding of circRNAs will be crucial for improving crop breeding and stress resistance.
(5) Conducting research on the application of circRNAs in plants: Further exploration of the potential applications of circRNAs, such as for plant genetic improvement, stress resistance breeding, and development of RNA pesticides, would provide new insights and methods for plant production and agricultural development.

In conclusion, further research is needed to explore the detailed mechanisms of action of circRNAs, functional validation, and interactions with other RNA regulatory factors. These efforts would help elucidate the complexity of plant gene regulatory networks. It is important to further research circRNAs for a deeper understanding of plant gene regulatory networks, deciphering plant stress response mechanisms, discovering new regulatory elements, and applying findings to plant improvement and breeding. Such studies would increase understanding of plant growth and development, and adaptation to the environment, thus providing a scientific basis for plant science and agricultural production, as well as valuable information for developing new control strategies.

## Figures and Tables

**Table 1:** Main research topics of circRNAs in this review.

Composition	Main Content				
Part 2	Classification of circRNAs	Part 2.1	Exonic circRNAs		
		Part 2.2	Intronic circRNAs		
		Part 2.3	Exon-intron circRNAs		
		Part 2.4	Intergenic circRNAs		
Part 3	Biogenesis of circRNAs	Part 3.1	Direct backsplicing model	Part 3.1.1	Intron pairing drives cyclization
				Part 3.1.2	Trans-acting factors drive cyclization
		Part 3.2	Exon jumping model		
Part 4	Interaction mechanisms of circRNAs with other biological macromolecules	Part 4.1	CircRNAs and their parental genes		
		Part 4.2	CircRNAs and miRNAs	Part 4.2.1	CircRNAs act as miRNA sponges
				Part 4.2.2	Predictive web tools for circRNA–miRNA interactions
				Part 4.2.3	Detection methods for circRNA–miRNA interactions
		Part 4.3	CircRNAs and proteins	Part 4.3.1	CircRNA–protein interactions
				Part 4.3.2	CircRNA–protein interactions prediction web tool
				Part 4.3.3	Detection methods for circRNA–protein interactions
		Part 4.4	CircRNAs and mRNAs		
Part 5	The physiological importance of circRNA in plants	Part 5.1	CircRNAs serving as miRNA sponges		
		Part 5.2	CircRNAs regulate gene expression		
		Part 5.3	Interactions between circRNAs and proteins		
		Part 5.4	CircRNAs have potential translation functions		
Part 6	Involvement of circRNAs in plant growth and development	Part 6.1	CircRNAs involved in plant embryogenesis		
		Part 6.2	Regulating plant nutritional growth via circRNAs		
		Part 6.3	Regulation of reproductive growth via circRNAs	Part 6.3.1	Regulation of flower development
				Part 6.3.2	Regulation of fruit development
Part 7	Roles of circRNAs in plant stress responses	Part 7.1	Responses of circRNAs to biostress	Part 7.1.1	CircRNAs and plant viral infections
				Part 7.1.2	CircRNAs and bacterial infections
				Part 7.1.3	CircRNAs and fungal infections
				Part 7.1.4	CircRNAs and insect attacks
		Part 7.2	CircRNA response to abiotic stresses	Part 7.2.1	CircRNAs and temperature stress
				Part 7.2.2	CircRNAs and water stress
				Part 7.2.3	CircRNAs and salt stress
				Part 7.2.4	CircRNAs and nutrient stress

**Table 2:** The functions of circRNAs and their corresponding research methods.

Order	Functions	Methods	References
1	CircRNAs regulate genes expression	CRISPR Cas9-guided promoter immunoprecipitation (CasIP) assay	[69]
		U1 antisense morpholino (AMO)	[37]
		R-loop dot-blotting, DNA–RNA immunoprecipitation and mass spectrum	[70]
2	CircRNAs serving as miRNA sponges	Luciferase reporter assays	[71]
		Antisense oligonucleotide (ASO) pulldown	[72]
		Labeled microRNA pulldown assays	[73]
		CircRNA–miRNA interaction assays	[74]
		RNA fluorescence in situ hybridization	[75]
		Silencing and overexpression experiments	[76,77]
3	Interactions between circRNAs and proteins	RNase protection assays	[52]
		RNA pulldown assays	[78]
		RNA immunoprecipitation	[79]
		Electrophoretic mobility shift assays	[80]
		Fluorescence in situ hybridization and immunofluorescence	[81,82]
4	CircRNAs have potential translation functions	m6A immunoprecipitation and quantification	[83]
		Ribosome footprinting (RFP) fragment sequencing experiments	[84]
		Nanopore-based direct RNA sequencing (DRS)	[85]
		Overexpression experiment	[86]

**Table 3:** Web tools for prediction of circRNA–miRNA interactions.

Database Name	Website ^a^	Sample Sources	Description	Refernces
Circ2Traits	http://mirtoolsgallery.tech/mirtoolsgallery/node/2155 (accessed on 19 June 2024)	Tissue samples from human diseases	Prediction of interactions between circRNAs and disease-associated miRNAs, construction of interaction networks and enrichment analysis methods for miRNAs and proteins, long non-coding RNAs, and/or circRNAs, and analysis of interaction sites between circRNA and SNPS of disease-associated genes	[99]
starBase v2.0	http://starbase.sysu.edu.cn/ (accessed on 19 June 2024)	Tissue samples from human diseases	Construction of miRNA–ceRNA, miRNA–ncRNA, and protein–RNA interaction networks from large-scale CLIP-Seq data	[100]
CircInteractome	https://circinteractome.nia.nih.gov/ (accessed on 19 June 2024)	Animal tissue samples	Information on RNA-binding proteins binding human circRNAs and miRNA binding sites, primer design tools, design of siRNAs for circRNA silencing, and identification of potential internal ribosome entry sites on circRNAs	[101]
CircNet	http://circnet.mbc.nctu.edu.tw/ (accessed on 19 June 2024)	Animal tissue samples	Newly identified circRNAs, integration of the networks of target genes of miRNAs, expression profiles of circRNA variable isoforms, genome annotations, and sequence information	[102]
AtCircDB	http://www.deepbiology.cn/circRNA/ (accessed on 19 June 2024)	*Arabidopsis*	Comprehensive tissue-specific database of circRNAs for *Arabidopsis*; retrieving, visualizing, and downloading circRNA data for *Arabidopsis*; and analysis of circRNA–miRNA interaction networks	[103]
PlantCircNet	http://bis.zju.edu.cn/plantcircnet/index.php (accessed on 19 June 2024)	*Arabidopsis*, rice, soybean, barley, tomato, wheat, maize, and purple falsebrome (*Brachypodium distachyon*)	Visualization tools for interaction network graphs; tools for GO enrichment analysis of overexpressed target genes of miRNAs; and information on the annotations of circRNA genomes, sequences, and shearsomes	[104]
PlantcircBase	http://ibi.zju.edu.cn/plantcircbase/ (accessed on 19 June 2024)	Rice, *Arabidopsis*, maize, tomato, and barley	Information on miRNA sponge function, circRNA–miRNA–mRNA interaction network graphs, tool for visualization of circRNA structure according to genome location, and tools for querying circRNA sequences	[105]
ASmiR	http://forestry.fafu.edu.cn/bioinfor/db/ASmiR (accessed on 19 June 2024)	Bamboo, rice, *Arabidopsis*, and eight other plants	Mutual regulation between target sites of miRNAs and linear RNAs, and between miRNAs and alternatively spliced circular RNAs	[106]
GreenCircRNA	http://greencirc.cn (accessed on 19 June 2024)	*Arabidopsis*, maize, rice, soybean, wheat, sunflower (*Helianthus annuus*), and sixty-three other plants	Database of plant circRNAs as miRNA decoys, a plant-based platform for exploration of plant circRNAs and their potential decoys	[107]
CircMiMi	https://github.com/TreesLab/CircMiMi (accessed on 19 June 2024)	Eighteen species (including sixteen animals and two plants)	A modular spftware based Python, that identifies circRNA–miRNA–mRNA interactions according to circRNA junction coordinates	[108]

^a^ This review is not responsible for the interpretation of the copyright issue of the tool, and readers are advised to contact the copyright owner to discuss the copyright issue.

**Table 4:** Web tools for prediction of circRNA–protein interactions.

Database Name	Website ^a^	Sample Sources	Description	References
catRAPID omics v2.0	http://service.tartaglialab.com/page/catrapid_omics2_group (accessed on 19 June 2024)	Eight model organisms (*Homo sapiens*, *Mus musculus*, *Rattus norvegicus*, *Xenopus tropicalis*, *Danio rerio*, *Drosophila melanogaster*, *Caenorhabditis elegans*, and *Saccharomyces cerevisiae*)	A web server dedicated to the computation of protein–RNA interaction propensities at the transcriptome- and RNA-binding proteome level in 8 model organisms. By integrating secondary structure, hydrogen bonding, and van der Waals forces, the web server accurately predicts protein–RNA binding effects.	[122]
CRMSS	https://github.com/BioinformaticsCSU/CRMSS (accessed on 19 June 2024)	Human disease tissue samples	Binding sites of circRNA-RBP are predicted, on the basis of multi-scale feature sequences and structural features.	[123]
CircSLNN	Offline software package (accessed on 19 June 2024)		RBP binding sites on circRNAs are identified by sequence labeling neural networks.	[124]
iCircRBP-DHN	https://github.com/houzl3416/iCircRBP-DHN (accessed on 19 June 2024)		A deep hierarchical network (DHN) is used to recognize circRNA-RBP binding sites.	[125]
RBPsuite	http://www.csbio.sjtu.edu.cn/bioinf/RBPsuite/ (accessed on 19 June 2024)		A deep learning-based online web server, RBPsuite, is used for predicting RBP binding sites on linear and circular RNAs.	[126]
CircRIP	https://github.com/bioinfolabwhu/circRIP (accessed on 19 June 2024)		RBP–circRNA interactions are systematically identified from RIP-Seq and eCLIP data.	[127]

^a^ This review is not responsible for the interpretation of the copyright issue of the tool, and readers are advised to contact the copyright owner to discuss the copyright issue.

## Data Availability

The data that support this study are available in the article.
